# The Oral Mouse Microbiome Promotes Tumorigenesis in Oral Squamous Cell Carcinoma

**DOI:** 10.1128/mSystems.00323-19

**Published:** 2019-08-06

**Authors:** Philip Stashenko, Susan Yost, Yoonhee Choi, Theodora Danciu, Tsute Chen, Subbiah Yoganathan, Christine Kressirer, Montserrat Ruiz-Tourrella, Bikul Das, Alexis Kokaras, Jorge Frias-Lopez

**Affiliations:** a Boston University Henry M. Goldman School of Dental Medicine, Boston, Massachusetts, USA; b Forsyth Institute, Cambridge, Massachusetts, USA; c Department of Periodontics and Oral Medicine, University of Michigan School of Dentistry, Ann Arbor, Michigan, USA; d Department of Cancer and Stem Cell Biology, Thoreau Lab for Global Health, University of Massachusetts—Lowell, Lowell, Massachusetts, USA; e Department of Oral Biology, University of Florida College of Dentistry, Gainesville, Florida, USA; University of California, Riverside

**Keywords:** OSCC, dysbiosis, metatranscriptome, microbiome, time series

## Abstract

Oral squamous cell carcinoma (OSCC) is the most common malignancy of the head and neck worldwide. Dysbiosis of the microbiome has increasingly been linked to the development of different kinds of cancer. Applying 16S rRNA gene sequence analysis and metatranscriptomic analyses, we characterized the longitudinal changes in the profiles and the function of the oral microbiome in a 4-nitroquinoline-1-oxide (4-NQO)-induced model of OSCC in gnotobiotic mice. We characterized the dynamics of the oral microbiome in this model using two different microbiome inocula: one from healthy mice and the other from mice bearing a 4-NQO-induced tumor. Mice colonized with different oral microbiomes and exposed to 4-NQO had increased tumor numbers and sizes compared to controls exposed to 4-NQO but lacking a microbiome. We observed an overall increase in diversity in the tumorigenic samples compared to that in the nontumor group not exposed to 4-NQO. Despite the variability in community dynamics, specific patterns emerged during the progression of the disease. In the two groups that were inoculated with the OSCC-associated microbiome, we observed opposite profiles of abundance in *Parabacteroides* and *Corynebacterium*. While the percentage of *Parabacteroides* bacteria decreased in the control group, it increased in the OSCC group, and the opposite was observed for *Corynebacterium*. The metatranscriptomic analysis revealed overexpression of the same metabolic signatures associated with OSCC regardless of the community profile. These included nitrogen transport, response to stress, interspecies interactions, Wnt pathway modulation, and amino acid and lipid biosynthesis. Thus, these results seem to suggest that certain collective physiological activities are critical for microbiome-mediated OSCC progression.

**IMPORTANCE** There is growing evidence that changes in the microbiome are associated with carcinogenesis. To date, no consistent oral microbiome composition associated with OSCC has been identified. Longitudinal and functional studies like the study presented here should yield a better understanding of the role that the oral microbiome plays in OSCC. Our findings, obtained using a germ-free mouse model, indicate that the presence of different oral microbiomes enhances tumorigenesis and increases the final number of tumors in mice. By studying community-wide expression profiles, we found that regardless of the phylogenetic composition of the microbiome, the same metabolic activities were consistently associated with OSCC. Therefore, due to the functional redundancy of the microbiome, the critical element in explaining the contribution of the microbiota in OSCC is the collective physiological activity of the community, thus accounting for the previous inability to identify a consensus community profile or etiologic agents for OSCC.

## Abstract (precis)

There is growing evidence that changes in the microbiome are associated with carcinogenesis. To date, no consistent oral microbiome composition associated with OSCC has been identified. Longitudinal and functional studies like the study presented here should yield a better understanding of the role that the oral microbiome plays in OSCC. Our findings, obtained using a germ-free mouse model, indicate that the presence of different oral microbiomes enhances tumorigenesis and increases the final number of tumors in mice. By studying community-wide expression profiles, we found that regardless of the phylogenetic composition of the microbiome, the same metabolic activities were consistently associated with OSCC. Therefore, due to the functional redundancy of the microbiome, the critical element in explaining the contribution of the microbiota in OSCC is the collective physiological activity of the community, thus accounting for the previous inability to identify a consensus community profile or etiologic agents for OSCC.

## INTRODUCTION

Oral squamous cell carcinoma (OSCC) is the most common malignancy of the head and neck, excluding nonmelanoma skin cancer. Worldwide, there were 300,000 cases of lip/oral cavity cancer (2.1% of the total number of cases of cancer worldwide) and 145,000 deaths from lip/oral cavity cancer (1.8% of the total number of deaths from cancer worldwide) in 2012 (1).

The two best-established etiologic factors in cancers of the oral cavity are alcohol and tobacco use. Besides, among infectious agents, human papillomavirus (HPV) has been identified to be an etiologic agent for oropharyngeal cancer in 2 to 4% of cases (2). However, many patients develop OSCC in the absence of these recognized risk factors. Although most studies assessing the infectious etiology of cancer have focused on viruses, recently, there has been increased interest in a possible role of the human bacterial microbiome in cancer (3, 4).

There were already indications that the oral microbiome may play a role in the tumorigenesis of OSCC. It is known that chronic periodontal disease is associated with an increase in the risk of development of OSCC (5–8). These observations have resulted in the hypothesis that the inflammatory microbiota associated with periodontitis may play a role in the development and progression of OSCC (9).

Moreover, members of the oral microbiome are capable of promoting various pathophysiologic functions that are associated with cancer. In a pioneer study, using a murine model of chronic infection-associated oral tumorigenesis in which experimental mouse periodontitis was induced by a combination of administration of Porphyromonas gingivalis and Fusobacterium nucleatum and administration of a specific oral carcinogen (4-nitroquinoline-1-oxide [4-NQO]), Binder Gallimidi et al. showed that these two prominent oral pathogens could promote tumor progression in mice (10). P. gingivalis infection has also been associated with orodigestive cancer (4), increased oral cancer invasion (11), and the proliferation of oral cancer stem cells (12).

Several studies have assessed the oral microbiome profiles associated with OSCC using molecular approaches. All of these studies observed a phenomenon referred to as dysbiosis, i.e., a significant shift in the composition of the oral microbiome in patients with OSCC from that in healthy controls. However, they found conflicting results regarding the composition and structure of the OSCC-associated microbiome (13–19). The factors leading to dysbiosis are complex and not well understood, and the effect of this abnormal community on OSCC remains unclear. In contrast to the findings obtained by analysis of the phylogenetic composition, more consistent and perhaps more informative results have been obtained with functional analysis rather than compositional analysis. Using Phylogenetic Investigation of Communities by Reconstruction of Unobserved States (PICRUSt) as a proxy to predict the oral microbiome functions (20), in two different studies, Perera et al. (17) and Al-Hebshi et al. (21) found that, despite the differences in community composition, certain results from functional prediction analysis were consistent between the two studies. Genes involved in bacterial motility, flagellar assembly, and bacterial chemotaxis synthesis were enriched in the tumors, and in particular, lipopolysaccharide (LPS) biosynthesis pathways were enriched in both cohorts. Moreover, in another independent study, genes related to protein and amino acid metabolism, such as valine, leucine and isoleucine, phenylalanine, tyrosine, and tryptophan biosynthesis, were inversely associated with OSCC progression (18). The same results were observed by Perera et al., where the genes responsible for phenylalanine, tyrosine, and tryptophan biosynthesis were significantly associated with the controls (17).

A more direct method to characterize community-wide gene expression profiles is metatranscriptomics, which is based on the set of transcripts synthesized by the microbial community under different conditions. This approach has been extremely informative in providing new insights into microbial functions and active communities in caries (22, 23), periodontitis (24–26), and gingivitis (27) and during biofilm formation and after meal ingestion (28). In a pilot study of community-wide gene expression analysis of the microbiome in OSCC, Yost et al. found that regardless of the community composition, specific metabolic signatures were consistently found in disease (16). Among them, metabolic signatures for iron ion transport, tryptophanase activity, peptidase activities, and superoxide dismutase were overrepresented in tumor and tumor-adjacent samples compared to samples from the healthy controls. The expression of putative virulence factors in the oral communities associated with OSCC showed that activities related to capsule biosynthesis, flagellum synthesis and assembly, chemotaxis, iron transport, hemolysins, and adhesins were upregulated at tumor sites.

In the current study, we used the well-established 4-NQO-induced carcinogenesis model of OSCC (29, 30) to determine the role of the oral microbiome in oral tumorigenesis, characterizing the dynamics of the oral microbiome and comparing the metatranscriptome of the final microbiome samples from mice exposed to the carcinogen with those from mice not exposed to the carcinogen.

## RESULTS

### The microbiome increases tumorigenesis in germfree mice.

**FIG 1:**
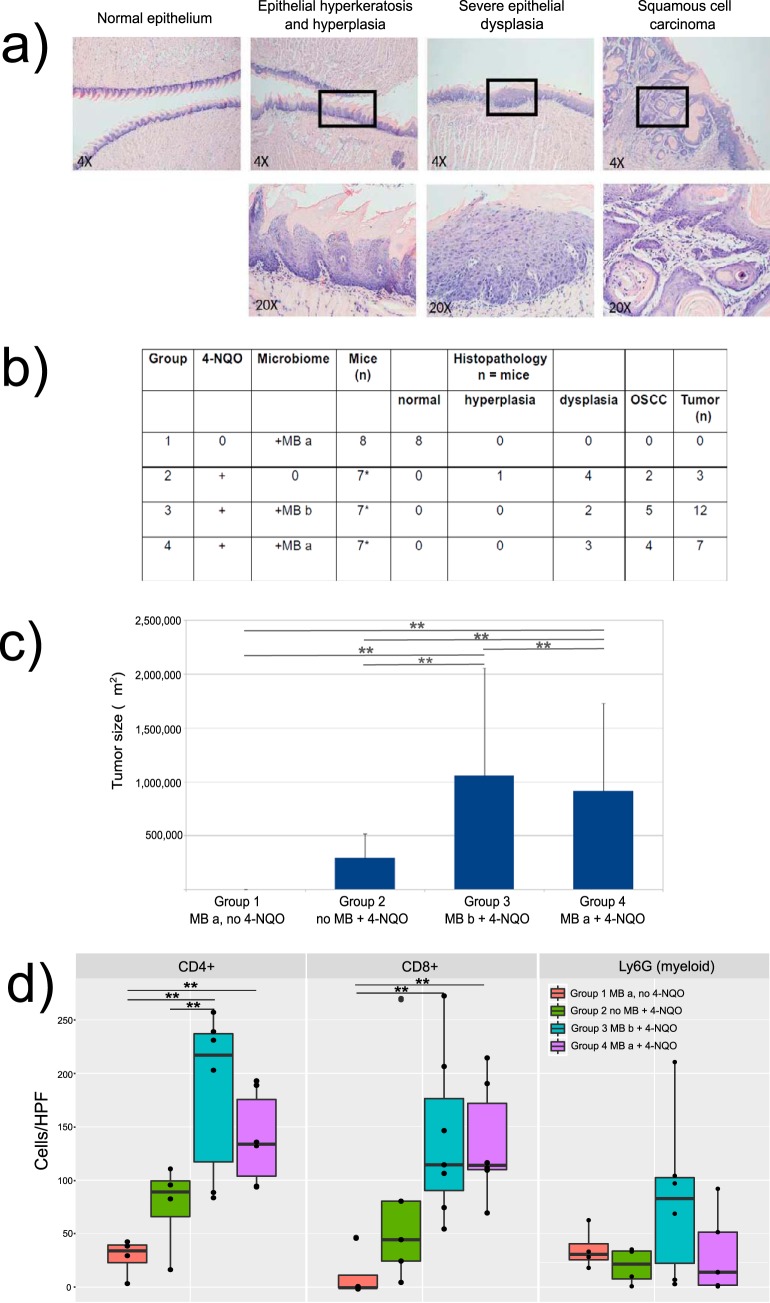
Oral squamous cell carcinoma (OSCC) induced by 4-nitroquinoline-1-oxide (4-NQO) in gnotobiotic mice. Mice were treated with 50 ppm of 4-NQO in the drinking water for 16 weeks, after which 4-NQO was withdrawn and the experiment was extended until 26 weeks. (a) (Top) Representative sections of tongues at week 26, showing normal, hyperplasia/hyperkeratosis, moderate dysplasia, and invasive OSCC. H&E staining. Magnification, ×40. (Bottom) Higher-power view (magnification, ×200) of the areas indicated by squares in the top row. (b) Summary of the experimental design and the number of mice with hyperplasia, various grades of dysplasia and OSCC in the different groups, as well as the number of tumors. MB a, inoculation with the murine microbiome (MB) from an OSCC-bearing mouse; MB b, inoculation with the microbiome from a healthy, tumor-free mouse. (c) Mean tumor size in the different experimental groups. (d) Cell infiltrates in sublesional areas in OSCC and dysplasia. Significant differences were calculated using the multiple comparison test after Kruskal-Wallis. *, see Fig. S1b in the supplemental material for *P* values for comparison of the number of tumors between the different groups; **, *P* < 0.05. HPF, high-power field.

We utilized the well-established 4-NQO-induced model of oral squamous cell carcinoma (OSCC) in germfree mice to determine the effect of the oral microbiome on tumorigenesis (see Fig. S1a in the supplemental material). As shown in Fig. 1, all mice exposed to 4-NQO in the drinking water for 16 weeks developed pathology, including precancerous lesions or OSCC, with a range of severity of dysplasia being seen in mice in groups 2, 3, and 4, whereas mice not given 4-NQO (but colonized with a microbiome) remained utterly free of pathology (group 1). Mice exposed to 4-NQO and colonized with an oral microbiome from either a healthy mouse (group 3) or an OSCC tumor-bearing mouse (group 4) developed more OSCC tumors than mice that were exposed to 4-NQO by that remained germfree (group 2) (Fig. 1b). The differences in the numbers of tumors and the frequencies of affected mice were statistically significant for all comparisons (Fig. 1c and Fig. S1b), regardless of the origin of the microbiome (Fig. 1b). All microbiome-free mice given 4-NQO (group 2) showed pathological changes in the tongue epithelium, but in most cases, these did not fully progress to invasive OSCC during the 26-week experimental period. Moreover, the tumors in mice colonized with a microbiome were significantly larger than those in mice lacking a microbiome (group 2; *P* < 0.05) (Fig. 1c and Fig. S1b). Unsuspectingly, among the 4-NQO-treated mice, the group colonized by the healthy microbiome (group 3) had more and bigger tumors than the group colonized by the OSCC-associated microbiome (group 4) (Fig. 1b and c and Fig. S1b).

10.1128/mSystems.00323-19.1 FIG S1 Histopathology of tongue epithelium in 4-NQO-treated mice with or without oral microbiomes. (a) Experimental timeline; red indicates 4-NQO treatment in drinking water. (b) Results for statistical differences for the number of tumors calculated, adjusting for multiple comparisons after the Kruskal-Wallis sum-rank test. FDR was 0.05. The table shows the adjusted *P* values for the different comparisons. Download FIG S1, PDF file, 0.02 MB. Copyright © 2019 Stashenko et al. 2019 Stashenko et al. This content is distributed under the terms of the Creative Commons Attribution 4.0 International license.

These results strongly indicate that the presence of an oral microbiome contributes to carcinogen-induced OSCC development, with the effects primarily being on tumor progression from precancerous dysplasia to invasive OSCC.

We also observed increased infiltration by host immune cells, including CD8^+^ cytotoxic T cells, CD4^+^ helper T cells, and Ly6G^+^ myeloid cells (neutrophils and monocyte/macrophages), into the sublesional areas of mice that received 4-NQO. CD8^+^ cytotoxic T cells and CD4^+^ helper T cells, including regulatory T cells, were significantly more numerous in mice treated with 4-NQO and inoculated with a microbiome (groups 3 and 4) than in the other groups (Fig. 1d). There was also a trend toward higher numbers of Ly6G^+^ cells, likely myeloid-derived suppressor cells (MDSC), in animals with the highest tumor burden (group 3; *P* was not significant) (Fig. 1d).

### Microbiome shifts associated with tumorigenesis.

**FIG 2:**
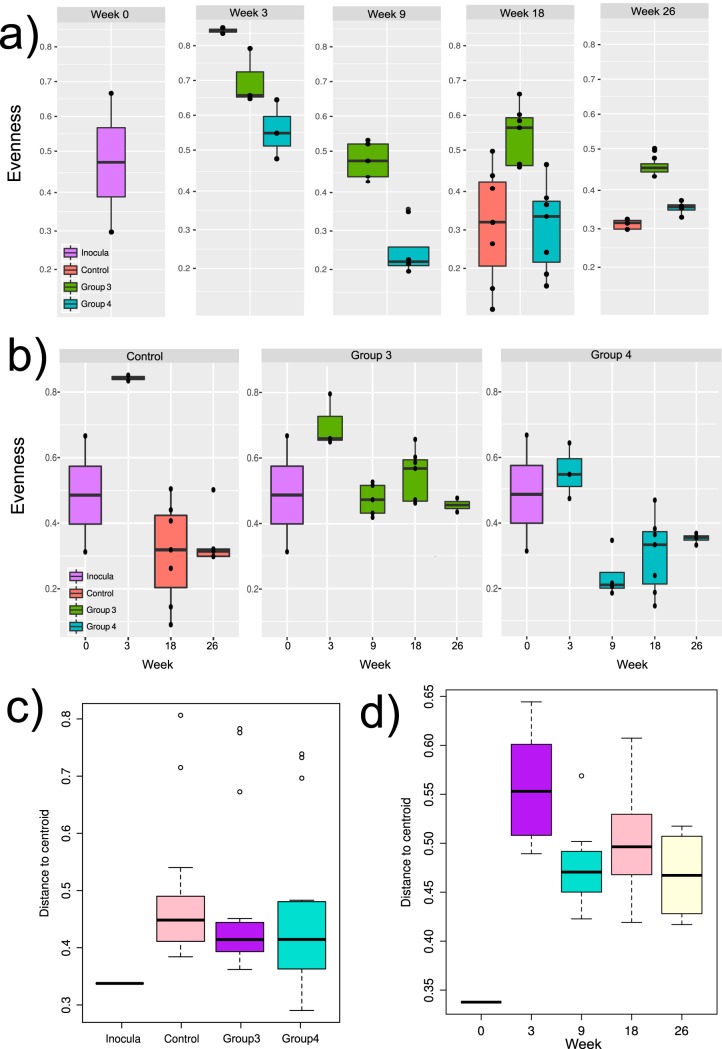
Changes in diversity in the different microbiomes. (a) Diversity measured as Pielou’s evenness (*J*′) index comparing the profiles by the week of the experiment. (b) Diversity measured as Pielou’s evenness index comparing the profiles by experimental group. (c) Box plots of beta diversity, measured as the average steepness (*z*) of the species-area curve in the Arrhenius model (*S* = *cX^z^*) comparing the profiles by experimental group. (d) Box plots of beta diversity, measured as the average steepness of the species-area curve in the Arrhenius model (*S* = *cX^z^*) comparing the profiles by the week of the experiment. Beta diversity is based on the ratio of total number of species in a collection of sites (*S*) and the average richness per one site. As described in the R package ‘vegan’ vignette, *X* is the size of the sample. Parameter *c* is uninteresting, but *z* gives the steepness of the species area curve and is a measure of beta diversity.

We examined the dynamics of the oral microbiome throughout the experimental period. Mice showed an initial increase in microbiome diversity after 3 weeks compared to the diversity in the inocula, followed by a significant decrease from this peak in microbial diversity, independent of the inoculum used (Fig. 2a and b). After 9 weeks, the microbiome from the group of mice inoculated with the health-associated microbiome and treated with 4-NQO (group 3) consistently showed a higher diversity than the microbiomes from the other two groups (Fig. 2a), which had been inoculated with the tumor-associated microbiome. Community-wide beta diversity analyses indicated that about 40% of the species were shared in the between-samples analysis (Fig. 2c). Interestingly, the number of shared species decreased after week 3 (Fig. 2d), indicating that different species were associated with the different groups of mice.

**FIG 3:**
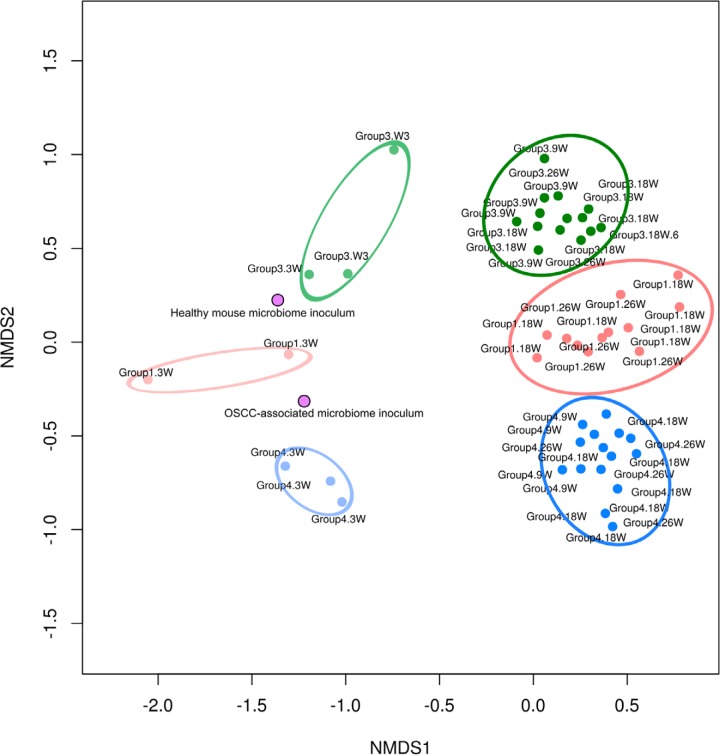
Nonmetric multidimensional scaling (NMDS) plot. Ordination is based on Brian-Curtis distances for all mice following colonization with a healthy microbiome or the microbiome from an OSCC-bearing mouse. Samples are circled according to the experimental group and week (W) sampled. Group 1, no 4-NQO treatment and inoculation with the murine OSCC-associated microbiome; group 3, 4-NQO treatment and inoculation with the murine health-associated microbiome; group 4, 4-NQO treatment and inoculation with the murine OSCC-associated microbiome.

Ordination of the distances between samples showed that after 3 weeks, there was a community shift and a clustering of the three different microbiomes associated with the three groups of mice (Fig. 3 and Fig. S2c). Beta diversities concerning classes or factors showed a similar pattern (Fig. 2d). The microbiomes were also separated by time of sampling. The profiles of the initial inocula and those at week 3 were similar, but they were still quite distinct from the more mature profiles at weeks 9, 18, and 26 (Fig. S2).

10.1128/mSystems.00323-19.2 FIG S2 Homogeneity of groups and beta diversity. Differences in beta diversities for classes or factors: groups (a), samples (b), and weeks (c). We used the betadisper function from the R package vegan. The plots indicate that there is a difference in species compositions based on groups (group 1, no 4-NQO treatment and inoculation with the murine OSCC-associated microbiome; group 3, 4-NQO treatment and inoculation with the murine health-associated microbiome; group 4, 4-NQO treatment and inoculation with the murine OSCC-associated microbiome) and the samples or weeks. Samples from week 3 and the initial inocula were more similar than samples from weeks 9, 18, and 26. Download FIG S2, PDF file, 0.1 MB. Copyright © 2019 Stashenko et al. 2019 Stashenko et al. This content is distributed under the terms of the Creative Commons Attribution 4.0 International license.

We next identified which operational taxonomic units (OTUs) were responsible for the shifts in the microbial community structure during tumorigenesis by analysis of 16S rRNA gene composition. Consistent with the results from our ordination of distances analyses, we observed significant differences in the microbiome composition at week 9 and the following time points analyzed but not at 3 weeks. At week 9, even before the tumors were observed, the experimental groups treated with 4-NQO and bearing a microbiome already presented significant differences that were maintained throughout the experiment (Fig. S3). Interestingly the differences between groups 3 and 4 decreased considerably at week 26, and only members of the families *Actinomycetaceae*, *Bifidobacteriaceae*, *Staphylococcaceae*, and *Clostridiaceae* were more abundant in group 3 than in group 4 (Fig. S3c).

10.1128/mSystems.00323-19.3 FIG S3 Differentially enriched bacteria in the two tumor samples. The results of linear discriminant analysis (LDA) effect size (LEfSe) analysis show bacteria that were altered between the OSCC and healthy controls. The analysis was performed on the 16S rRNA gene sequencing results. Histograms report th**e** taxa showing different abundance values between group 3 and group 4 at 9, 18, and 26 weeks. The alpha value for the Kruskal-Wallis (KW) sum-rank test was 0.1, and that for the Wilcoxon test was 0.05. Only taxa with an LDA of >3 are represented in the cladograms. (a) Nine weeks; (b) 18 weeks; (c) 26 weeks. Group 1, no 4-NQO treatment and inoculation with the murine OSCC-associated microbiome; group 3, 4-NQO treatment and inoculation with the murine health-associated microbiome; group 4, 4-NQO treatment and inoculation with the murine OSCC-associated microbiome. Download FIG S3, PDF file, 0.6 MB. Copyright © 2019 Stashenko et al. 2019 Stashenko et al. This content is distributed under the terms of the Creative Commons Attribution 4.0 International license.

**FIG 4:**
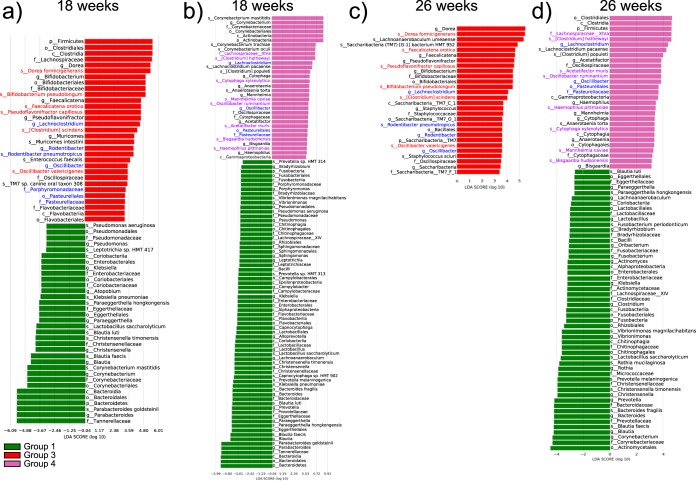
Bacteria differentially enriched in tumor samples compared to the bacteria in the healthy controls. The results of linear discriminant analysis (LDA) effect size (LEfSe) analysis show bacteria that were altered between the OSCC and healthy controls. The analysis was performed on the 16S rRNA gene sequencing results. Histograms report the taxa showing a different abundance of values between group 1 (the control) and groups 3 and 4 at 18 and 26 weeks. The alpha value for the Kruskal-Wallis (KW) sum-rank test was 0.1, and that for the Wilcoxon test was 0.05. Only taxa with an LDA of >3 are represented in the cladograms. (a) Group 1 versus group 3 at 18 weeks; (b) group 1 versus group 4 at 18 weeks; (c) group 1 versus group 3 at 26 weeks; (d) group 1 versus group 3 at 26 weeks. Group 1, no 4-NQO treatment and inoculation with the murine OSCC-associated microbiome; group 3, 4-NQO treatment and inoculation with the murine health-associated microbiome; group 4, 4-NQO treatment and inoculation with the murine OSCC-associated microbiome.

Pairwise comparisons against the control group (group 1) showed that in both tumor groups, members of the family *Pasteurellaceae* and genera *Lachnoclostridium* and *Oscillibacter* were consistently present in significantly higher numbers in the tumor sites than in the controls after week 18 (Fig. 4). Mice colonized with the OSCC-associated microbiome consistently showed a significant increase in Acetatifactor muris, Bisgaardia hudsonensis, Cytophaga xylanolytica, Mannheimia caviae, Oscillibacter ruminantium, and Haemophilus pittmaniae (Fig. 4). In contrast, mice colonized with the health-associated microbiome (group 3) showed a significant increase in the presence of Bifidobacterium pseudolongum, [*Clostridium*] *scindens*, Dorea formicigenerans, Faecalicatena orotica, Oscillibacter valericigenes, and Pseudoflavonifractor capillosus. (The NCBI Taxonomy staff places square brackets around the genus for some species to indicate that they are misclassified, meaning placed incorrectly in a higher taxonomic rank. The species is awaiting to be formally renamed through the appropriate Code of Nomenclature, but until then the incorrect genus is indicated by the square brackets.) Additionally, we observed in this group an increase in Rodentibacter pneumotropicus, a member of the family *Pasteurellaceae* (Fig. 4). These results strongly suggest that tumorigenesis is associated with dysbiosis of the oral microbiome, as highlighted by significant shifts in bacterial populations from a wide range of taxonomic groups.

### Time series 16S rRNA gene sequence analysis of the microbiome during tumorigenesis.

**FIG 5:**
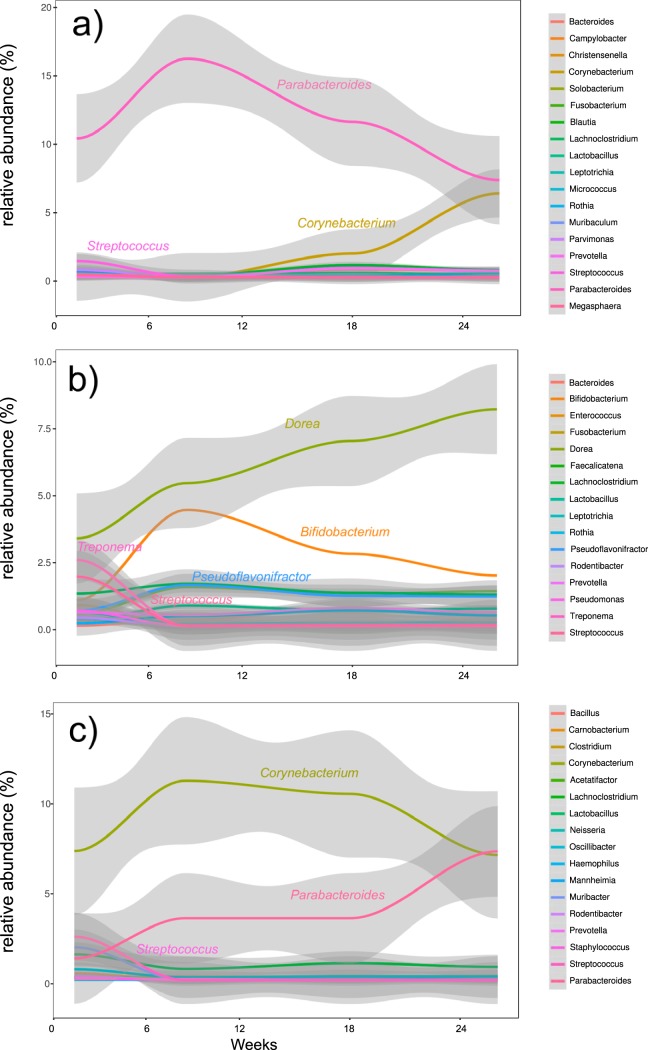
Relative abundance trajectories of the different genera during the duration of the experiment. The graphs show the relative abundance (*y* axis) of the most common bacterial genera over time (*x* axis). Shaded regions indicate 95% confidence intervals. (a) Group 1 (no 4-NQO treatment and inoculation with the murine OSCC-associated microbiome; (b) group 3 (4-NQO treatment and inoculation with the murine health-associated microbiome); (c) group 4 (4-NQO treatment and inoculation with the murine OSCC-associated microbiome).

Studies of these ecosystems over time require specific analytic approaches to explore their dynamics and identify signatures relevant to the outcomes. We performed a time series analysis of our 16S rRNA gene sequence analysis results using the R package splinectomeR. Our results showed opposite trajectories of two genera in the communities which were colonized with the OSCC-associated microbiome. Mice not treated with 4-NQO showed an initial high percentage of members of the genus *Parabacteroides* and a low percentage of members of the genus *Corynebacterium* (Fig. 5a) at the beginning of the experiment. However, over time, the *Parabacteroides* fraction decreased, while the *Corynebacterium* fraction increased (Fig. 5a). In contrast, the group of mice inoculated with the same OSCC-associated microbiome but treated with 4-NQO showed the complete opposite trajectories of these two genera. While *Parabacteroides* was present at a low frequency at 3 weeks, its relative abundance increased with time, whereas *Corynebacterium* had the opposite trajectory (Fig. 5c). The microbial communities from mice inoculated with a health-associated microbiome (group 3) showed a completely different profile. The genus *Dorea* increased in relative abundance throughout the experiment, and *Bifidobacterium* increased during the first 9 weeks, followed by a slight decrease after that (Fig. 5b). The same longitudinal analyses at the species level showed that Parabacteroides goldsteinii and Corynebacterium mastitidis were the most significant contributors to the profiles observed at the genus level in mice colonized with the OSCC-associated microbiome (Fig. S4a and c). In contrast, in the health-associated microbiome, the species D. formicigenerans and B. pseudolongum showed trajectories that mimicked the genus profiles (Fig. S4b).

10.1128/mSystems.00323-19.4 FIG S4 Relative abundance trajectories of the different species during the duration of the experiment. The graphs show the relative abundance (*y* axis) of the most common bacterial species over time (*x* axis). Shaded regions indicate 95% confidence intervals. (a) Group 1 (no 4-NQO treatment and inoculation with the murine OSCC-associated microbiome); (b) group 3 (4-NQO treatment and inoculation with the murine health-associated microbiome); (c) group 4 (4-NQO treatment and inoculation with the murine OSCC-associated microbiome). Download FIG S4, PDF file, 0.1 MB. Copyright © 2019 Stashenko et al. 2019 Stashenko et al. This content is distributed under the terms of the Creative Commons Attribution 4.0 International license.

While they are informative, Fig. 5 and Fig. S4 do not represent the results of any statistical analysis but instead present a visual representation of the behavior of the different taxonomic units over time. We used a different set of functions from the splinectomeR package to test whether the abundance of a taxonomic unit (genus and species) was significantly different across the time series between the control microbiome not exposed to 4-NQO and the two 4-NQO-exposed microbiomes. Table S1 shows the significance of the differences between the trajectories of different genera and species across the time series. Despite the differences described above, specific patterns of behavior with significant differences were conserved in both comparisons: group 1 versus group 3 and group 1 versus group 4. For instance, the genus *Parabacteroides* showed a tendency to decrease in relative abundance in the control group, while *Lachnoclostridium* showed a tendency to decrease in relative abundance in the 4-NQO-exposed tumorigenic microbiomes, and *Corynebacterium*, *Blautia*, and *Christensenella* increased in relative abundance with time (Fig. S5). The time series analysis by species showed an increase in relative abundance for D. formicigenerans, [*Clostridium*] *scindens*, Faecalicatena orotica, and B. pseudolongum in group 3, while only A. muris showed a significant increase in relative abundance in group 4 (Fig. S6). Interestingly, the results of linear discriminant analysis (LDA) effect size (LEfSe) analysis for the different time points showed D. formicigenerans to be a biomarker of group 3 (Fig. 4).

10.1128/mSystems.00323-19.5 FIG S5 Permuted spline tests for statistical significance in longitudinal microbiome data. The permuspliner output plot shows that the difference between genus abundance between the control group (group 1; group spline in red) and group 3 (a) and group 4 (b) (group spline in blue). The translucent lines depict the permuted splines, which serve as a visual for how distinct the two groups are relative to the random assortment. Download FIG S5, PDF file, 0.3 MB. Copyright © 2019 Stashenko et al. 2019 Stashenko et al. This content is distributed under the terms of the Creative Commons Attribution 4.0 International license.

10.1128/mSystems.00323-19.6 FIG S6 Permuted spline tests for statistical significance in longitudinal microbiome data. The permuspliner output plot shows that the difference between species abundance between the control group (group 1) (group spline in red) and group 3 (a) and group 4 (b) (group spline in blue). The translucent lines depict the permuted splines, which serves as a visual for how distinct the two groups are relative to the random assortment. Download FIG S6, PDF file, 2.9 MB. Copyright © 2019 Stashenko et al. 2019 Stashenko et al. This content is distributed under the terms of the Creative Commons Attribution 4.0 International license.

10.1128/mSystems.00323-19.9 TABLE S1 Statistical significance of the differences in trajectories for the 20 most abundant genera and species. The table shows the FDR-adjusted *P* values (*q* values) of the different tests performed. Download Table S1, PDF file, 0.04 MB. Copyright © 2019 Stashenko et al. 2019 Stashenko et al. This content is distributed under the terms of the Creative Commons Attribution 4.0 International license.

### Active communities are very similar, regardless of total community composition, based on 16S rRNA gene profiles.

**FIG 6:**
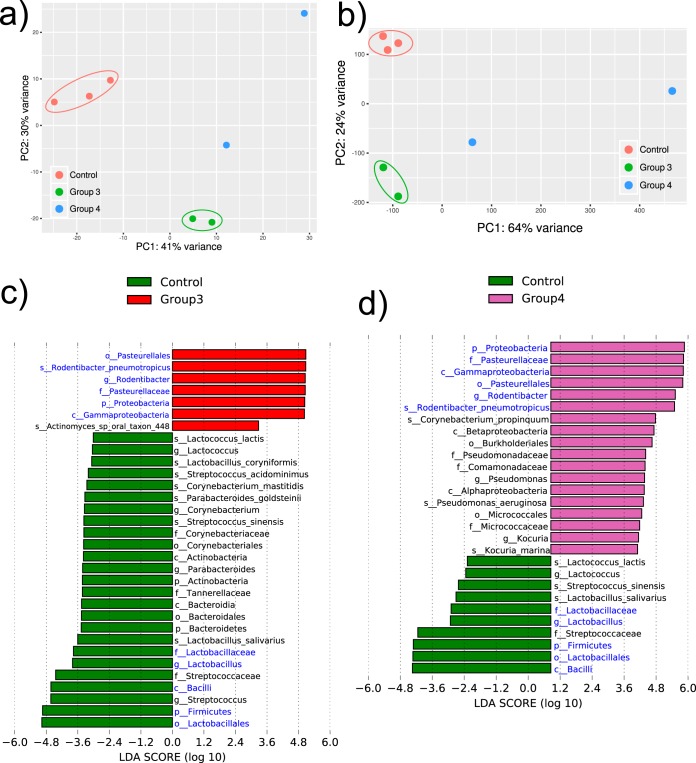
Statistical differences in the phylogenetic composition of different active microbiomes and community-wide expression profiles. Metatranscriptome hit counts were obtained by comparison of the sequences against an in-house mouse oral microbiome database using the Kraken algorithm. (a) PCA based on the phylogenetic composition of the active communities based on metatranscriptomic results (Kraken profiles). (b) PCA based on the raw community-wide expression profiles of the different samples. (c) Histogram of the LDA scores computed for features by linear discriminant analysis (LDA) effect size (LEfSe) analysis, showing bacteria that were altered between group 1 (control) and group 3. Green, control (no 4-NQO treatment and inoculation with the murine OSCC-associated microbiome); red, group 3 (4-NQO treatment and inoculation with the murine health-associated microbiome). (d) Histogram of the LDA scores computed for features by linear discriminant analysis effect size analysis showing bacteria that were altered between group 1 (control) and group 4. Green, control (no 4-NQO treatment and inoculation with the murine OSCC-associated microbiome); purple, group 4 (4-NQO treatment and inoculation with the murine OSCC-associated microbiome). The alpha value for the Kruskal-Wallis (KW) sum-rank test was 0.1, and that for the Wilcoxon test was 0.05. Only taxa with an LDA of >3 are represented. Blue, taxa that were also identified to be altered using the HUMAnN2 pipeline (see Fig. S7 in the supplemental material).

We then proceeded to analyze the metatranscriptome of the communities at the termination of the experiment on week 26. We first mapped our transcriptome sequencing results against a custom mouse oral microbiome database that we generated, thus obtaining the profiles of active (mRNA-expressing) members of the community. Interestingly, when we performed principal-component analysis (PCA) of the gene expression profiles, we observed a distribution similar to the one obtained for the phylogenetic composition of the active communities, with a distinct separation of the three communities (Fig. 6a and b). However, the microbiomes of two samples from group 4 used for metatranscriptome analysis showed more considerable differences in mRNA compositions and active communities than the microbiomes of the other two groups (Fig. 6a and b).

10.1128/mSystems.00323-19.7 FIG S7 Linear discriminant analysis (LDA) effect size (LEfSe) analysis of phylogenetic assignments of the metatranscriptome using the HUMAnN2 pipeline. The phylogenetic assignments were obtained from the results obtained with the MetaPhlAn2 database, included in the HUMAnN2 pipeline. (a) Histogram of the LDA scores computed for features by LEfSe analysis showing bacteria that were altered between group 1 (control) and group 3. (b) Histogram of the LDA scores computed for features by LEfSe analysis showing bacteria that were altered between group 1 (control) and group 4. Green, control group (no 4-NQO treatment and inoculation with the murine OSCC-associated microbiome); red, group 3 (4-NQO treatment and inoculation with the murine health-associated microbiome); purple, group 4 (4-NQO treatment and inoculation with the murine OSCC-associated microbiome). The alpha value for the Kruskal-Wallis (KW) sum-rank test was 0.1, and that for the Wilcoxon test was 0.05. Only taxa with an LDA of >3 are represented. Blue, taxa that were also identified to be altered using the Kraken algorithm (see Fig. 6). Download FIG S7, PDF file, 0.3 MB. Copyright © 2019 Stashenko et al. 2019 Stashenko et al. This content is distributed under the terms of the Creative Commons Attribution 4.0 International license.

We then characterized the active communities using two different algorithms, Kraken and the results obtained with the MetaPhlAn2 database, which is included in the Human Microbiome Project Unified Metabolic Analysis Network (HUMAnN2) pipeline. No significant differences were observed when we compared the two groups from tumor samples using either Kraken or the HUMAnN2 pipeline, indicating that the active communities in tumor samples at the end of the experiment were not significantly different. When comparing group 1 (the control group) and groups 3 and 4, we observed commonalities using both algorithms. First, *Pasteurellales* and, more specifically, members of the genus *Rodentibacter* (R. pneumotropicus) were always highly active in both cancer groups (Fig. 6c and d and Fig. S7). There were also minor specific differences. For instance, members of the genus *Pseudomonas* (Pseudomonas aeruginosa) were active in group 4 but not in group 3. The fact that the same results were observed using both Kraken and MetaPhlAn2 (included in the HUMAnN2 pipeline) underscores the robustness of these results, even though the Kraken library is a custom mouse library, while the MetaPhlAn2 library was the default library from the software and not specific to the oral mouse microbiome. Likewise, the control group showed similarities to both tumorigenic microbiome-inoculated groups. The genus *Lactobacillus* was always identified to be more active in Kraken and HUMANnN2, while the genus *Parabacteroides* and, more specifically, Parabacteroides goldsteinii were identified to be more active in group 4 than in group 3, as determined using both the Kraken and MetaPhlAn2 algorithms.

### Common overrepresented metabolic activities in tumorigenic microbiomes are independent of community composition.

We then proceeded to analyze the metatranscriptome of the communities at the termination of the experiment on week 26, comparing the expression profiles of the control group (group 1) with those of groups 3 and 4, which were inoculated with tumorigenic microbiomes. We used two different strategies to perform the functional analysis of the metatranscriptome.

**FIG 7:**
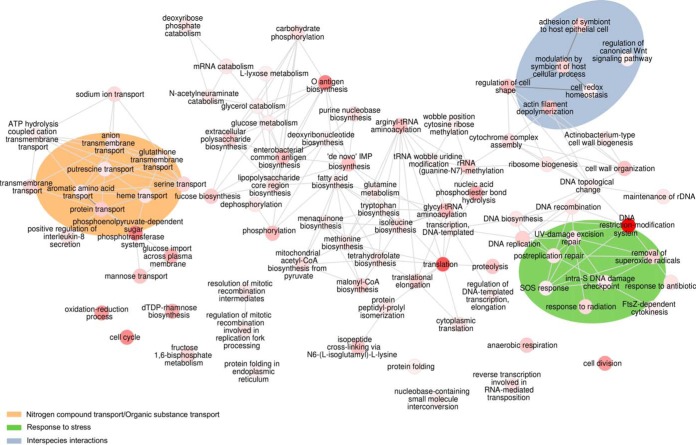
GO enrichment analysis for the metatranscriptome profiles of the oral mouse microbiome associated with cancer status. Overrepresented biological processes are indicated. Enriched terms were obtained using GOseq, and the terms were summarized and visualized as a network using the REVIGO webpage. The bubble color indicates the user-provided *P* value (the value for darker red is closer to 0). Edges in the graph link highly similar GO terms. CoA, coenzyme A; rDNA, rRNA gene.

We first performed a community-wide differential expression analysis, using a genome database derived from our results from 16S rRNA analysis, described above (Table S2). Differential expression analysis performed using this database identified 3,243 differentially expressed (DE) genes between the control group not treated with 4-NQO and group 3 and 1,892 DE genes between the control group and group 4 (Fig. S8). Of those DE genes, 1,845 were differentially expressed in both analyses (Fig. S8). Therefore, there appeared to be a core pool of DE genes associated with OSCC progression, regardless of the overall community composition. More importantly, when we performed Gene Ontology (GO) enrichment analysis, we obtained the same exact profile of enriched GO terms, whether we analyzed the DE genes from the control group versus group 3 comparison or the control group versus group 4 comparison (Fig. 7). These results indicate that the functional activities in the tumorigenic microbiomes are independent of the phylogenetic composition of the communities. Three specific clusters of activities were highly represented, including nitrogen/organic substance transport, response to stress, and functions associated with interspecies interactions (Fig. 7). There were also a large number of overrepresented activities involved in cell wall biosynthesis (e.g., O-antigen, lipopolysaccharide core region, and enterobacterial common antigen biosynthesis) and amino acid metabolism (glutamine, isoleucine, and tryptophan biosynthesis). The oxidative stress response of the microbiome was also highly overrepresented, with GO terms such as SOS response, response to radiation, removal of superoxide radicals, and UV damage excision repair being among the activities associated with the OSCC microbiomes (Fig. 7). Other biological processes overrepresented were proteolysis, which has previously been linked to virulence in periodontal disease (24, 25), and anaerobic respiration, which may indicate a highly anaerobic environment in the OSCC site (Fig. 7).

10.1128/mSystems.00323-19.8 FIG S8 Enriched functions in the metatranscriptome, determined using the HUMAnN2 pipeline. The summary of the linear discriminant analysis (LDA) effect size (LEfSe) analysis shows pathways that were significantly differentially enriched between the cases and controls (LDA score ≥ 3) in our metatranscriptome analysis. The analysis is based on MetaCyc pathway definitions (76), detected with the HUMAnN2 pipeline. Sixty-six pathways were enriched in both groups 3 and 4. Nine pathways were enriched only in group 3, and five were included only in group 4. The Venn diagram shows the percentage of common and specific pathways to the two tumor groups. Download FIG S8, PDF file, 0.2 MB. Copyright © 2019 Stashenko et al. 2019 Stashenko et al. This content is distributed under the terms of the Creative Commons Attribution 4.0 International license.

10.1128/mSystems.00323-19.10 TABLE S2 Genomes used in the mouse oral microbiome database. It contains 2,894 genomes from 526 species of *Bacteria*. Download Table S2, XLS file, 0.1 MB. Copyright © 2019 Stashenko et al. 2019 Stashenko et al. This content is distributed under the terms of the Creative Commons Attribution 4.0 International license.

*N*-Acetylneuraminic acid is the most abundant sialic acid found in the cell membranes of eukaryotes, and we observed an increase in activities associated with *N*-acetylneuraminate catabolism. The bacterial genes associated with this activity included those for *N*-acetylneuraminate lyase, glucosamine-6-phosphate deaminase, *N*-acetylglucosamine-6-phosphate deacetylase, *N*-acetylmannosamine kinase, *N*-acetylmannosamine-6-P epimerase, and *N*-acetylneuraminate lyase. We also observed an overrepresentation of activities associated with the regulation of the canonical Wnt signaling pathway, and concordantly, we also observed an increase in activities associated with actin filament depolymerization (Fig. 7).

**FIG 8:**
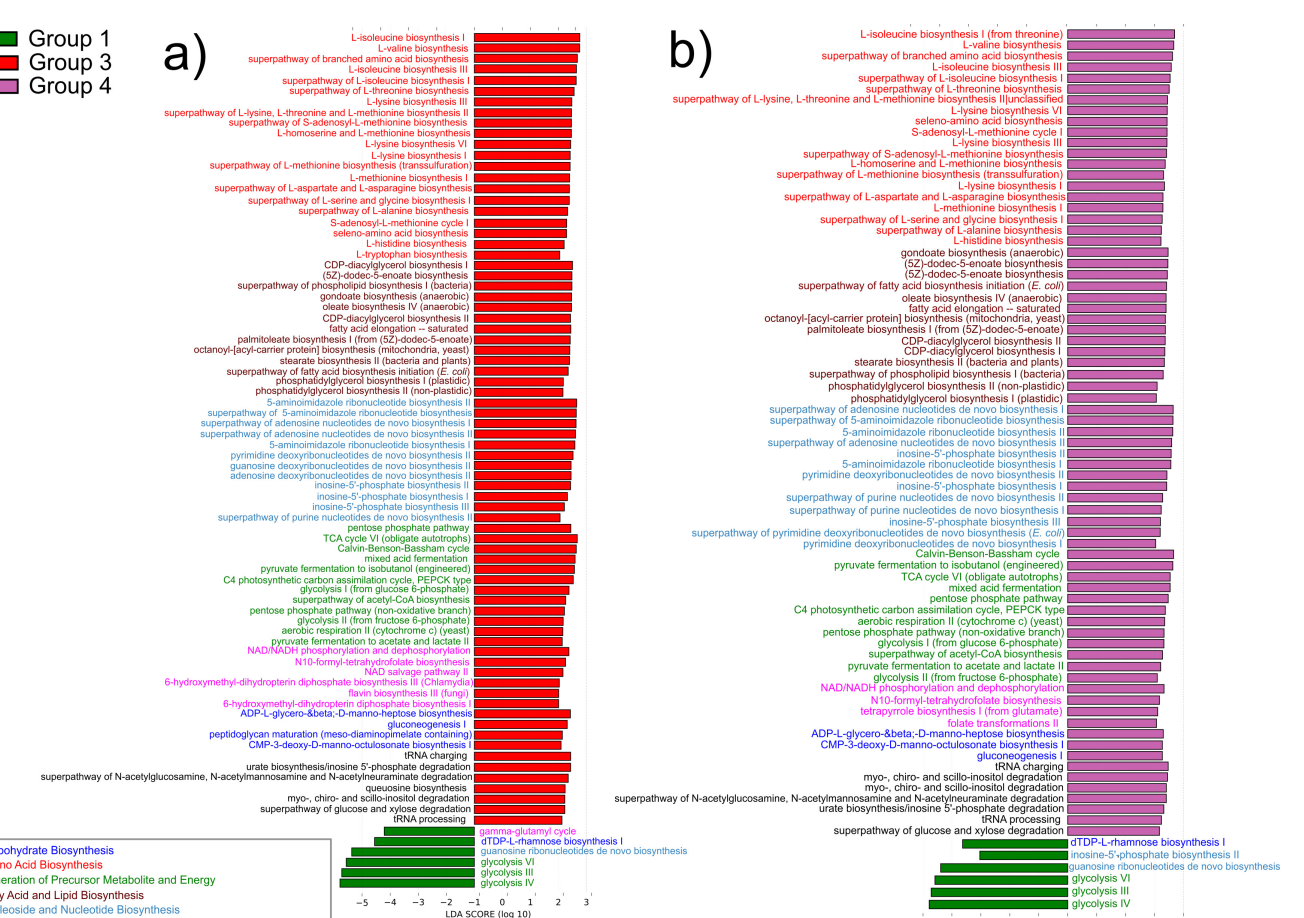
Differentially enriched functions in the metatranscriptome. Linear discriminant analysis (LDA) effect size (LEfSe) analysis showing pathways that were significantly differentially enriched between the cases and the controls (LDA score ≥ 3) in our metatranscriptome analysis. The analysis is based on MetaCyc pathway definitions (77), detected with the HUMAnN2 pipeline. Different pathway classes have different colors and are ranked from the most abundant to the least abundant. (a) Group 1 versus group 3; (b) group 1 versus group 4. Group 1, no 4-NQO treatment and inoculation with the murine OSCC-associated microbiome; group 3, 4-NQO treatment and inoculation with the murine health-associated microbiome; group 4, 4-NQO treatment and inoculation with the murine OSCC-associated microbiome. TCA, tricarboxylic acid; PEPCK, phosphoenolpyruvate carboxykinase.

The second strategy for differential functional abundance analysis between the controls and tumor samples was to use the HUMAnN2 pipeline. In this case, the results are presented as MetaCyc database-based pathway definitions instead of GO terms. Consistent with the results of the enrichment analysis of GO terms, most of the enriched pathways were in the tumorigenic microbiome-inoculated groups (Fig. 8). Moreover, as with the GO enrichment analysis, most pathways present in the tumor samples were the same, regardless of the community composition. Eighty-two percent (66 pathways) were observed in both comparisons (Fig. 8), while only 9 pathways were specific to group 3 and only 5 pathways were specific to group 4. The same was true for the control group compared to the tumorigenic microbiome-inoculated groups. Five out of six pathways enriched in the control group were common to both comparisons against groups 3 and 4 (Fig. 8). The most significant fraction of pathways in the tumorigenic microbiome-inoculated groups was associated with amino acid biosynthesis. Three other large groups of pathways were enriched in tumor sites: lipid biosynthesis, nucleoside and nucleotide biosynthesis, and generation of energy pathways (Fig. 8).

## DISCUSSION

In the present study, we established a causal role for the oral mouse microbiome in exacerbating OSCC development in a murine model of carcinogen-induced tumorigenesis. The transfer of microbiomes from either tumor-bearing or healthy tumor-free mice into germfree recipients significantly increased the numbers and sizes of tumors compared to those in mice that remained germfree but that were exposed to 4-NQO. Studies on the microbial composition associated with human OSCC have demonstrated differences in microbial composition between cancerous and healthy tissues (31, 32). However, there have been inconsistent and contradictory reports in the literature regarding which specific bacteria or patterns of oral microbial dysbiosis are consistently implicated in OSCC (13, 33).

Although we demonstrated dynamic changes in the microbiome during tumorigenesis, the differences between the murine and human systems must be considered before translating these results to humans. The oral microbiomes of mice and humans are very different (34, 35), with mice being coprophagic, hence the high number of intestinal organisms commonly present in the oral mouse microbiome (34).

Although many bacterial taxa were altered in relative abundance throughout the experiment, it is as yet unclear whether those changes are the result of tumorigenesis or are the causal agents of increased pathogenesis. Regardless, we observed a significant increase in the numbers and sizes of tumors in mice in which microbiomes were present compared to those in the germfree controls. These increases could be the result of an increase in the level of inflammation in the tumor microenvironment (36, 37), although other microbe-derived signals are capable of modulating hallmarks of cancer by other mechanisms independent of the control of inflammation (e.g., sustaining proliferative signaling, increasing genome instability, and mutation rates) (38). Using a murine model of colorectal cancer, Zackular et al. demonstrated that inoculation of germfree mice with a tumor-associated microbiome resulted in tumors greater in number and larger than those in mice inoculated with a microbiome from healthy tumor-free mice (39). Notably, in our case, regardless of the origin of the inocula, we observed an increase in the numbers and sizes of the tumors. This difference could be because the oral and colorectal systems represent very different environments, and besides, Zackular et al. (39) repeatedly inoculated the mice with fecal material, while in the present study, mice were colonized only by the initial inoculum, with no repetitions.

We observed at the end of the experiment an overall increase in diversity in the tumorigenic samples compared to that in samples from the nontumor group not exposed to 4-NQO. An increase in microbial diversity has also been observed in human OSCC in association with the progression stage of the disease (18) and in other types of carcinomas (33). Despite the variability in community dynamics, specific patterns emerged during the progression of the disease. In the two groups that were inoculated with the OSCC-associated microbiome, we observed opposite profiles of relative abundance in two genera: *Parabacteroides* and *Corynebacterium*. While the percentage of bacteria of the genus *Parabacteroides* decreased in the control group, it increased in the OSCC group, and the opposite was observed for *Corynebacterium*. Decreased carriage of the genus *Corynebacterium* has been described in patients with esophageal squamous cell carcinoma (ESCC) (40), and a greater abundance of this genus has been associated with a decreased risk of head and neck squamous cell cancer (HNSCC) (41). In colorectal cancer, it has been reported that some *Parabacteroides* species are enriched in human gut carcinoma samples compared with healthy and adenoma samples (33). Moreover, Parabacteroides distasonis attenuates colonic inflammation and tumorigenesis in mice (42, 43) and can reduce inflammation by inducing the anti-inflammatory cytokine interleukin-10 (IL-10) and suppressing the secretion of inflammatory cytokines IL-17, IL-6, and gamma interferon (44).

Inflammation plays an essential role in tumorigenesis, and chronic inflammation increases cancer risk (38, 45). In general, we observed a clear correlation between pathology and the numbers of immune cells infiltrated and a trend toward a possible more immunosuppressive infiltrate in the group inoculated with the murine health-associated microbiome, which had the most tumors, than in the group inoculated with the murine OSCC-associated microbiome. Additionally, we observed a decrease in members of the genus *Bifidobacterium* in the group inoculated with the health-associated microbiome and a concordant increase in the members of the genus *Dorea*. In the colon, *Bifidobacterium* interacts with butyrate-producing colon bacteria, favoring their coexistence (46). Bifidobacteria, however, do not produce butyrate, leaving the mechanism of enhanced butyrate production unresolved, although bacterial cross feeding likely plays a role (47). These bacterial populations may serve as protective mediators of oral health in the murine microbiome by stimulating the production of butyrate, which has been shown to reduce inflammation (48, 49) and induce apoptosis in a variety of tumor cell lines (50, 51). In the case of *Dorea*, an increase in its frequency in epithelial precursor lesions in patients with oral cancer compared to healthy subjects has been described (14), though its possible role in tumorigenesis is unknown.

Our metatranscriptomic results support the hypothesis that different microbial communities can promote tumorigenesis in an oral murine model of OSCC, which is strongly reinforced by our data showing no differences in the metabolic profiles of the two OSCC microbiomes, although their composition and time series behavior were entirely different. Nonetheless, in our analysis of active communities, Rodentibacter pneumotropicus (formerly Pasteurella pneumotropica) was highly active in both communities in tumors. R. pneumotropicus is a member of the family *Pasteurellales* isolated predominantly from rodents (52). R. pneumotropicus is one of the most important infectious agents in laboratory animals, with a reported prevalence of 4% to 13% in Europe and North America, respectively (53).

One of the surprising results from our experiments is the fact that mice inoculated with the health-associated microbiome yielded more and larger tumors than the group inoculated with the OSCC-associated microbiome. We do not have a clear explanation for these results; nonetheless, R. pneumotropicus was enriched in group 3 in comparison to group 4 at week 18. If R. pneumotropicus is vital for the tumorigenesis process, it means that it had colonized group 3 before it did group 4, giving the health-associated microbiome a head start in the development of the disease.

Specific functional changes in the microbiome, for example, lipopolysaccharide (LPS) production, polyamine synthesis, butyrate metabolism, and oxidative phosphorylation, could be linked to colon cancer (54). We found that in our metatranscriptomic enrichment analysis, LPS production, phosphorylation, and polyamine (putrescine) transport were all overrepresented in the tumor-promoting microbiome samples. An increase in LPS production is probably associated with the inflammatory microenvironment ideal for tumor progression (39, 54), while high levels of putrescine have been reported in human OSCC samples (55, 56). In two previous studies, LPS production was identified to be a potentially important activity in the OSCC microbiome in humans (17, 21).

We also detected an overrepresentation of metabolic activities associated with antioxidant systems, indicating that the OSCC microenvironment is highly enriched in reactive oxygen species (ROS). Many studies have documented the role of ROS in both the initiation and the promotion of multistep carcinogenesis. Oxidative damage to cellular DNA leads to mutations and may play an essential role in the initiation and progression of carcinogenesis in OSCC (57–59). In a pilot study comparing the metatranscriptomes of healthy versus OSCC-associated microbiomes in human cancer, we also found that metabolic activities associated with antioxidant systems were enriched in the tumor sites (16).

Furthermore, we observed activities associated with interspecies interactions, some of which suggest the possibility of the direct modulation of tumorigenesis. Two biological processes overrepresented in the tumorigenic microbiomes are directly linked to the modulation of host cells: activities associated with *N*-acetylneuraminate catabolism and activities associated with the regulation of the canonical Wnt signaling pathway, the latter of which were overrepresented.

The aberrant behavior of the canonical Wnt signaling pathway is a common feature of many tumors and plays essential roles in tumor progression and the metastasis of many cancer types (60, 61). Aberrant signaling of the Wnt/β-catenin signaling pathway may also be an essential element in the process of tumorigenesis that leads to malignancy in OSCC (61, 62). Our results indicate that the microbiome is involved in the process of regulating the canonical Wnt signaling pathway. The actual mechanism is unclear, but this raises the exciting hypothesis that tumorigenic microbiomes directly modulate a critical pathway linked to the progression of malignancy.

The failure to identify a consensus community profile or etiological agent is likely due to the considerable variation in the structure of the microbiome across individuals and the improbability that there is only a single community profile or bacterial population that is associated with OSCC progression. Moreover, microbiomes with different compositions may express similar functions. Thus, microbiomes with different compositions could render the same outcomes. There is a growing recognition that the composition of the microbiome may not be as crucial as the function of the microbiome in maintaining host health (17, 21, 63, 64). Our results support that conclusion, since we found that, regardless of the overall composition and dynamics of the oral community, the same metabolic activities were consistently found to be overrepresented in the microbiomes associated with OSCC, further suggesting that, rather than the microbiome composition *per se*, it is the collective physiological activity of the community that is important in disease.

## MATERIALS AND METHODS

### Study design, animals and animal care, and sample collection.

Germfree Swiss Webster male mice (8 weeks old) were obtained from Taconic Biosciences, Rensselaer, NY. Mice were maintained under gnotobiotic conditions in isolators in the Forsyth Institute Germ-Free Mouse Facility. Mice were fed autoclaved chow and water *ad libitum*. All animal experiments were approved by the Forsyth Institute Institutional Animal Care and Use Committee.

Mice were randomized into four groups (*n* = 8), as follows: (i) a group that was not treated with 4-nitroquinoline-1-oxide (4-NQO) and that was inoculated with a healthy mouse microbiome (group 1); (ii) a group that was treated with 4-NQO and that was not inoculated with a microbiome (group 2); (iii) a group that was treated with 4-NQO treatment and that was inoculated with an OSCC-associated mouse microbiome (group 3); and (iv) a group that was treated with 4-NQO and that was inoculated with healthy mouse microbiome (group 4). To minimize cage effects, each treatment group was housed in two separate cages. The microbiome used as the inoculum was sampled using cotton swabs that were rubbed against the surface of the tongue of mice of the same strain used in our experiments and resuspended in artificial saliva medium (65) that had been prereduced for 4 days in an anaerobic chamber. The sources for the inocula were healthy mouse tongues and tumor sites from mice that had already developed OSCC in a previous experiment. Mice were inoculated with the indicated microbiomes on day 0 by oral gavage and swabbing on the paws and then exposed to 4-NQO at 50 ppm in the drinking water. After 16 weeks, 4-NQO was withdrawn and replaced with regular water, to minimize the development of esophageal tumors (66), until the termination of the experiment at 26 weeks.

### Sample collection.

Swab samples of the oral microbiome were taken at 1, 3, 9, 18, and 26 weeks, as indicated in Fig. S1a in the supplemental material. Each sample was placed in individual tubes containing 0.5 ml of RNAlater (Life Technologies, Grand Island, NY, USA) and stored frozen at −80°C. At the end of the experiment, additional samples were taken for metatranscriptome analysis and stored as described above. A total of seven samples (three from group 1 [controls], two from group 3, and two from group 4) were used for metatranscriptome analysis.

### Microbiome RNA and DNA extractions.

RNAlater was gently removed from the tubes containing the swab. DNA and RNA were extracted simultaneously. For RNA extraction, mirVana kit lysis/binding buffer (600 μl) and 0.1-mm zirconia-silica beads (300 μl; BioSpec Products) were added to the samples. The beads were cleaned and sterilized beforehand with a series of HCl acid and bleach washes, treated with diethyl pyrocarbonate overnight, and autoclaved. Samples were bead beaten for 1 min at maximum speed in mirVana isolation kit lysis buffer, followed by performance of the manufacturer's instructions for the mirVana isolation kit (Life Technologies). At this point, the DNA contained in the phenolic fraction was extracted following the protocol described in the instructions provided by the manufacturer of the ToTally RNA kit (Life Technologies).

For metatranscriptome analysis, a MICROB*Enrich* kit (Life Technologies) was used to remove eukaryotic RNA and a MICROB*Express* kit (Life Technologies) was used to remove prokaryotic rRNA, in both cases following the manufacturer’s instructions.

### RNA amplification and Illumina sequencing.

RNA amplification was performed on total enriched bacterial RNA using a MessageAmp II-Bacteria RNA amplification kit (Life Technologies) following the manufacturer’s instructions. Sequencing was performed at the Forsyth Institute Sequencing Core. Illumina adapter-specific primers were used to amplify and selectively enrich for the cDNA generated from enriched mRNA. A TruSeq Stranded mRNA kit was used to generate libraries from amplified DNA. Samples were run using a NextSeq 500 sequencer and a 2 × 75-bp 150-cycle (v2) reagent kit (Illumina). The only variation to the original Illumina protocol is that the protocol for the samples began at the end of the purification and fragmentation of the mRNA by adding approximately 400 ng in 5 μl to 13 μl of the fragment primer finish mix.

### 16S rRNA sequence data analysis.

The 16S rRNA next-generation sequencing (NGS) was performed at the Forsyth Institute (Cambridge, MA). PCR amplification of DNA (10 to 50 ng) was performed using universal primers targeting the V3-V4 region of 16S rRNA genes (primers F341 and R806). The products were purified using AMPure purification. Amplicons were pooled in libraries (100 ng) that were gel purified and quantified by quantitative PCR before being sequenced (MiSeq sequencer; Illumina, San Diego, CA). In this study, reads were typically >50,000 per sample. The sequence read pairs were merged to single reads with a script (join_paired_ends.py) provided by the Quantitative Insights into Microbial Ecology (QIIME) package (v1.91) (67) with the default settings. The merged reads were then taxonomically assigned to the species level on the basis of a published algorithm (68). Briefly, merged sequence reads were searched, using the BLASTN program, against a panel of full-length 16S rRNA sequences that consisted of 998 sequences from the Human Oral Microbiome Database (HOMD) RefSeq database (v15.1), 495 sequences from the HOMD RefSeqExtended database (v1.1), 3,940 sequences from GreenGeneGold database, and 19,670 sequences from the NCBI 16S rRNA reference database. After the taxonomy assignment, species-level operational taxonomic units (OTUs) with at least 10 reads were subject to several downstream bioinformatics analyses, including alpha (evenness) and beta diversity assessments, as well as linear discriminant analysis effect size (LEfSe) (69) for differential microbiome OTU analysis.

We used the *betadiver* function from the R package vegan for beta diversity calculation (70) and nonmetric multidimensional scaling (NMSD) analysis. Pielou's measure of species evenness was calculated using the *evenness* function from the R package asbio. Finally, we performed performing linear discriminant analysis (LDA) effect size (LEfSe) as proposed by Segata et al. (69) with default parameters but an LDA cutoff of 3.

### Metagenome time series analysis.

16S rRNA gene sequencing results were used for time series analysis. Given that we had some missing values in our time series, we used the ts and na.interpolation functions from the imputeTS R package (71) to impute them. We performed the cubic (or Hermite) spline interpolation on the individual profiles of each species in the different groups. We then used the R package splinectomeR (72) to perform comparisons in our longitudinal data. The longitudinal comparisons were performed at two taxonomic levels: genus and species. The function *permuspliner* allows for the testing of statistical significance of the longitudinal data.

### Taxonomic profiles based on the metatranscriptomes.

Counts from the mRNA libraries were used to determine their phylogenetic composition for bacteria and archaea. The phylogenetic profiles of the metatranscriptomes were obtained using the Kraken algorithm (73). We generated a custom Kraken library with the oral microbiome genomes indicated in Table S2. The phylogenetic profiles were used to identify significant differences between active communities under the different conditions studied by performing linear discriminant analysis (LDA) effect size (LEfSe), as proposed by Segata et al. (69), by using an LDA cutoff of 3 and a Kruskal-Wallis significance of 0.1. Additionally, we analyzed the taxonomic profiles obtained using the MetaPhlAn2 database from the HUMAnN2 analysis (see below). Taxa differentially abundant between groups were identified through the use of LEfSe with the same parameters described above.

### Functional analysis.

We performed two types of functional analyses on the metatranscriptomes. One used the HUMAnN2 pipeline and is based on the analysis of gene families, while the other was based on the enrichment of Gene Ontology (GO) terms associated with the genes of a custom database generated from the species identified in our 16S rRNA gene sequencing results that we obtained with downloaded genomes belonging to 746 strains of bacteria that cover different 510 species (Table S2).

### HUMAnN2 analysis.

The gene family abundance, pathway abundance, and pathway coverage of each sample were determined directly from processed reads using the Human Microbiome Project Unified Metabolic Analysis Network (HUMAnN2) pipeline (v0.11.2) with default parameters (74, 75). HUMAnN2 utilizes the UniRef90 (76), MetaCyc (77), and MinPath (78) databases combined with the MetaPhlAn2 and ChocoPhlAn pangenome databases to characterize the taxa, genes, and pathways present in sequenced data sets. The nucleotide-level and translated searches were accelerated by running the Bowtie2 and Diamond (79) programs, respectively. We focused our analysis on the output of pathway abundance, which provided comprehensive quantitative insight into the functional aspects of a microbial community. Differentially abundant pathways between groups were identified through LEfSe with the same parameters described above.

### GO enrichment analysis.

Genomes, protein sequences, and gff files were downloaded using ncbi-genome-download scripts (https://github.com/kblin/ncbi-genome-download). Low-quality sequences were removed from the query files. Fastx clipper and the fastq quality filter from the Fastx-toolkit (hannonlab.cshl.edu/fastx_toolkit/) were used to save short sequences with a quality score of >20 in >80% of the sequence. Cleaned sequences were then aligned against sequences in the database using the Bowtie2 program with the parameters -q –local -N 1 -L 20 -D 30 -t -R 3 -i S,1,0.25, as described by Duran-Pinedo et al. (24). Sequence Alignment/Map (SAM) results were converted to the BAM format and sorted using the SAMtools program suite (80). Read counts from the SAM files were obtained using the BEDtools *multicov* tool from the BEDtools suite (81).

Differentially expressed genes from the RNA libraries were identified using the R package DESeq2 (82).

To evaluate functional activities differentially represented in our samples, we mapped the differentially expressed genes to known biological ontologies based on the Gene Ontology (GO) project (http://www.geneontology.org/). GO terms associated with the different proteins in our database were obtained using the program blast2GO (83). Briefly, we downloaded the Reviewed Swiss-Prot database (uniprot_sprot.fasta) from UniProt (https://www.uniprot.org/downloads) and used the BLAST+ executables from NCBI to format the database with the *makeblastdb* tool. We compared our protein sequences against those in the uniprot_sprot.fasta database with the BLAST program and the following command; ./blastx -db ∼/path/to/your/myformattedDBname/-outfmt 5 -evalue 1e-3 -word_size 3 -show_gis -num_alignments 20 -max_hsps 20 -num_threads 5 -out local_blast.xml -query myquery.fasta. Blast2GO uses those BLAST analysis results in xml format to map and annotate the proteins as GO terms.

Enrichment analysis of these sets was performed using the R package GOseq, which accounts for biases due to the overdetection of long and highly expressed transcripts (84). We used the REVIGO webpage (85) to summarize and remove redundant GO terms from the results. Only GO terms with false discovery rates (FDR) of <0.05 were used. REVIGO plots were obtained for two categories (biological process and molecular function). In the case of specific organisms, we mapped upregulated genes to GO terms and ranked them before summarizing the results using REVIGO. The plots were visualized either using the R script obtained from REVIGO or the network xmmgl file, which can be opened and modified in the Cytoscape (v3) software platform (86).

### Histopathological analysis of tissue samples.

Mice were sacrificed by CO_2_ inhalation after 26 weeks. Tongues were isolated, fixed in 4% paraformaldehyde (Sigma-Aldrich), embedded in paraffin (VWR), sectioned at 7 μm, and stained with hematoxylin and eosin (H&E). The histological assessment was performed by a board-certified pathologist (T.D.) in a blind manner. Histopathological findings were classified as normal mucosa, hyperkeratosis and epithelial hyperplasia (benign lesions), dysplasia (further subclassified as mild, moderate, or severe), and oral squamous cell carcinoma (OSCC; malignant) (Fig. 1b). The diagnostic classification was based on 2005 WHO guidelines for typing squamous cell carcinoma and precancerous lesions of the oral mucosa, as previously described (87). The criteria for normal mucosa included a surface stratified squamous epithelium of normal thickness without hyperplasia or hyperkeratosis and with overlying connective tissue being mostly devoid of inflammation. Benign lesions included those with hyperkeratosis and/or epithelial hyperplasia without cellular atypia or disordered maturation. Epithelial dysplasia was reserved for those cases with cellular atypia and disordered maturation encompassing the basal third of the epithelium (mild), extending from the basal layer to the midportion of the epithelium (moderate), or extending from the basal layer to above the midpoint of the epithelium (severe). Features indicative of cellular atypia included prominent nucleoli, an increased nuclear-to-cytoplasmic ratio, dyskeratosis, and increased and/or abnormal mitotic figures. A diagnosis of squamous cell carcinoma was rendered when islands and cords of malignant squamous epithelial cells arising from dysplastic surface epithelium invaded the adjacent connective tissue stroma.

For each animal, every 10th section was evaluated separately. The histopathological diagnosis for each section was based on the site with the most severe epithelial disturbances. For example, as illustrated in Fig. 1, a specific section contained areas classified as mild, moderate, and severe dysplasia; the final histopathological diagnosis for this section was severe epithelial dysplasia. A final diagnosis for each animal was obtained as follows: normal mucosa when all examined sections demonstrated normal mucosa, the most frequently noted histopathological change for benign lesions (hyperkeratosis and/or hyperplasia) or dysplasia, and the number of separate sites for squamous cell carcinoma. The histopathological findings of the study are summarized in Fig. 1b. Tumor size was determined by histomorphometry as a cross-sectional area through the center of each lesion using ImageJ software.

### Immunohistochemistry.

Infiltrating immune cells were identified using rat monoclonal antibodies against CD4 (helper T cells), CD8a (cytotoxic T cells), and Ly6G (neutrophils, macrophage/monocytes) or normal rat IgG (control; catalog number 6-001-A), all of which were from R&D Systems (Minneapolis, MN). Tongue sections were treated with citrate buffer, pH 6.0, and microwaved to unmask hidden epitopes, followed by overnight incubation with antibodies at 4°C in triplicate. Sections were washed 5 times in phosphate-buffered saline–0.1% Tween 20 (Sigma-Aldrich), and endogenous peroxidase was blocked with 0.3% peroxide hydrogen in methanol (Sigma-Aldrich) for 20 min at room temperature. Primary antibodies were detected using a Vectastain Elite ABC HRP kit (Vector Laboratories) according to the manufacturer’s instructions, counterstained with Fast Green (Sigma-Aldrich), and mounted with Permount mounting medium (Fisher Scientific). Images were acquired with a laser-scanning confocal system (Zeiss 780). Image analysis was performed with ZEN 2009 software (Zeiss) over at least three high-power fields (HPF). Data are expressed as the mean number of positive cells per HPF ± standard deviation.

### Data availability.

The data sets used in these analyses were deposited at the Human Oral Microbiome Database (HOMD; http://www.homd.org/ftp/publication_data/20180530/). The raw sequence reads used in this study are available in NCBI's Sequence Read Archive (SRA) under BioProject accession number PRJNA549752.
