# Supplementary figures and images for: The Oral Mouse Microbiome Promotes Tumorigenesis in Oral Squamous Cell Carcinoma
Source: mSystems. 2019 Aug 6;4(4):e00323-19. doi: 10.1128/mSystems.00323-19 (PMC6687944; doi:10.1128/mSystems.00323-19)

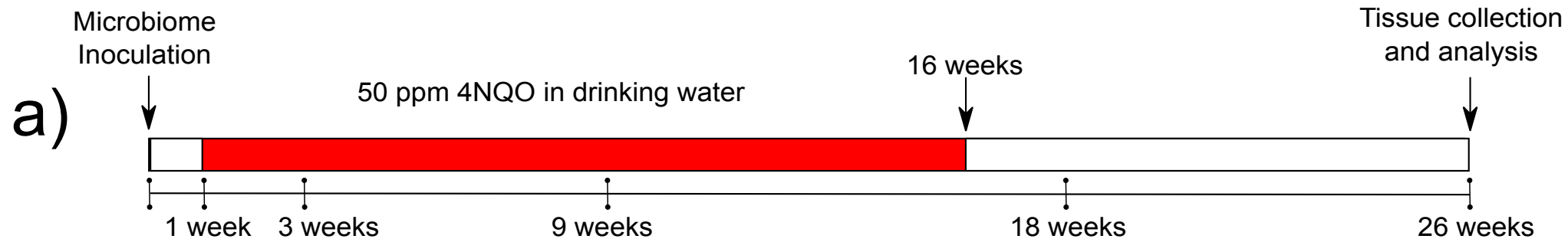

b)

|                   | Difference | p-value |
|-------------------|------------|---------|
| Group 1- Group 2  | -5.142857  | 0.0136  |
| Group 1- Group 3  | -18.071429 | 0.0000  |
| Group 1- Group 4  | -12.000000 | 0.0000  |
| Group 2 - Group 3 | -12.928571 | 0.0000  |
| Group 2 - Group 4 | -6.857143  | 0.0032  |
| Group 3 - Group 4 | 6.071429   | 0.0067  |

Supplement: FIG S1 [file mSystems.00323-19-sf001.pdf]

a)

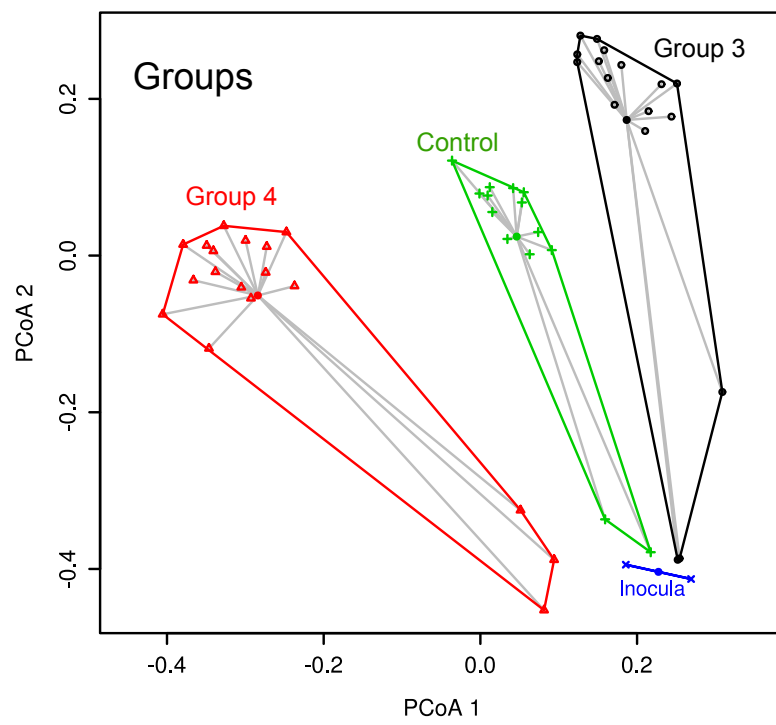

b)

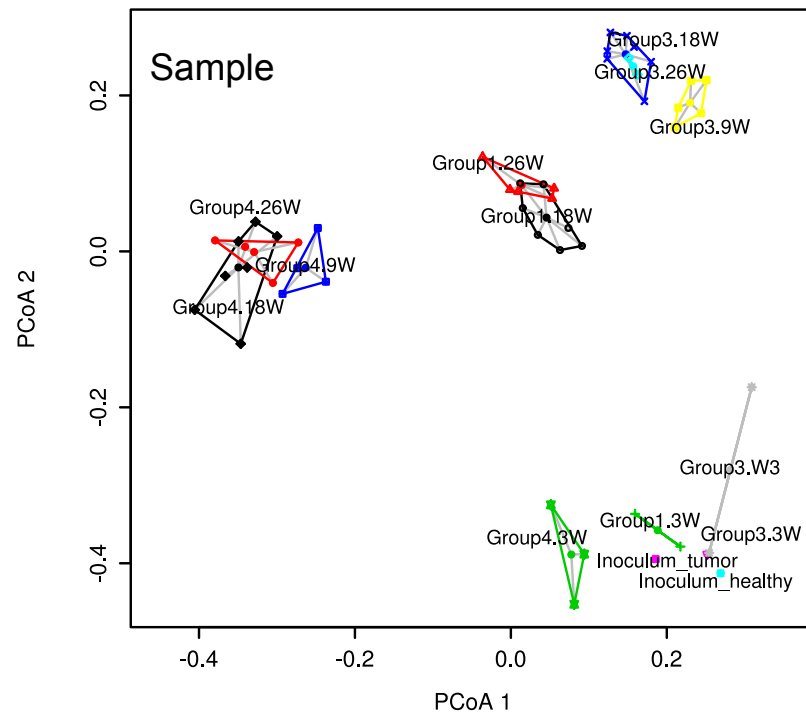

c)

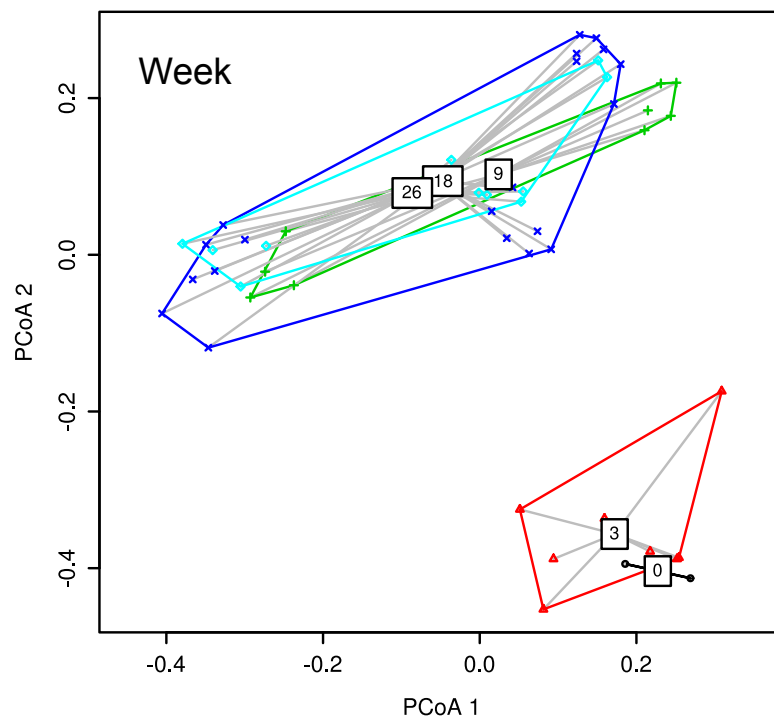

Supplement: FIG S2 [file mSystems.00323-19-sf002.pdf]

Group 3  
Group 4

a)

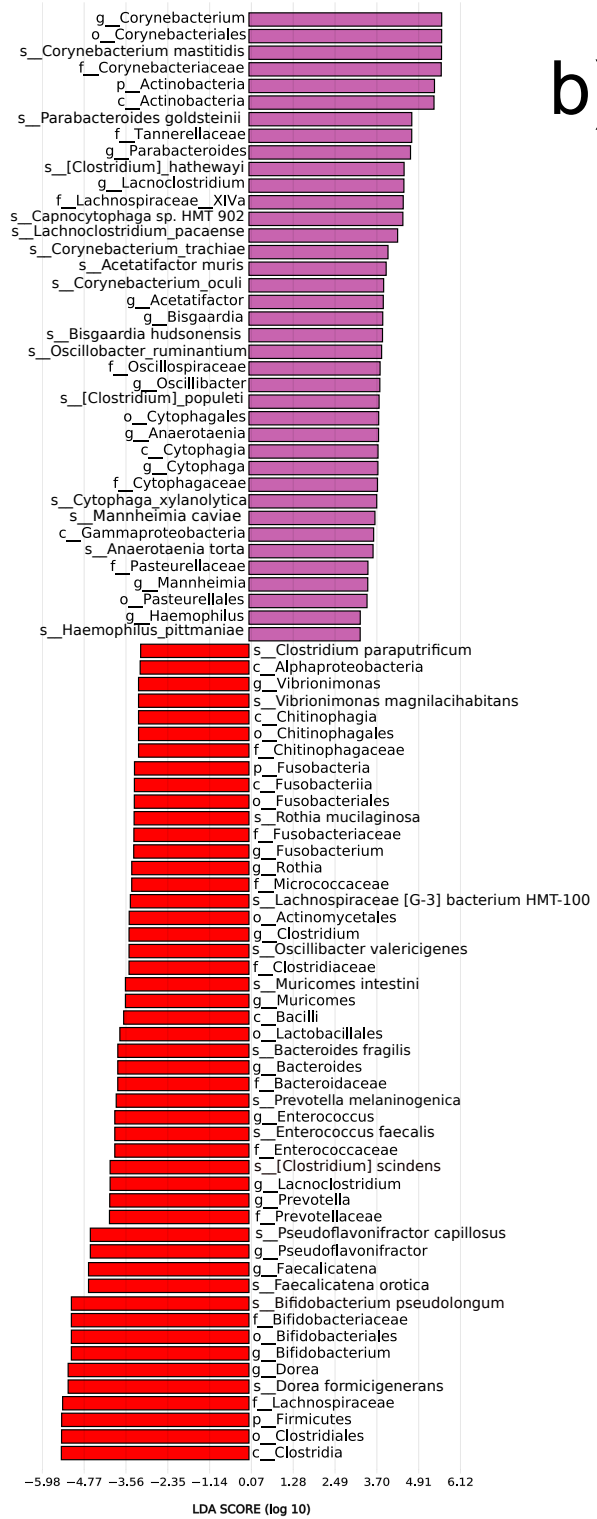

b)

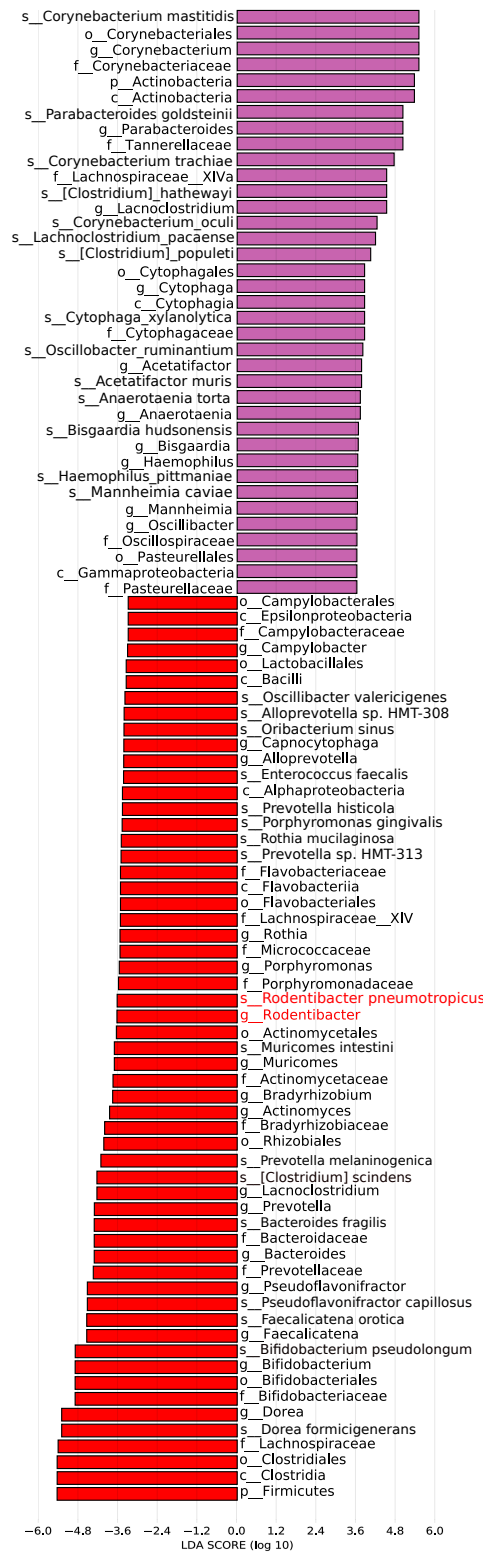

c)

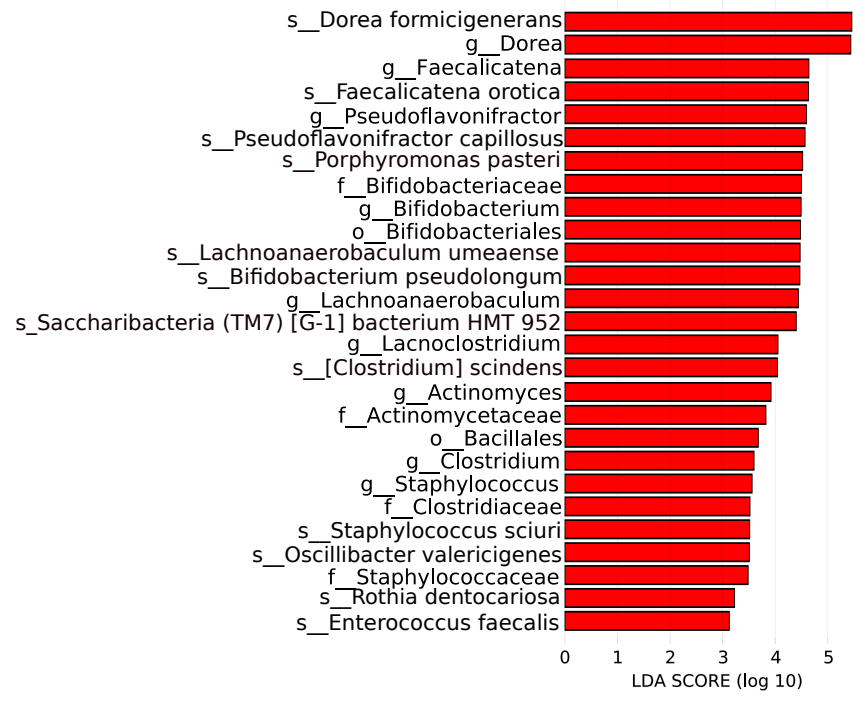

Supplement: FIG S3 [file mSystems.00323-19-sf003.pdf]

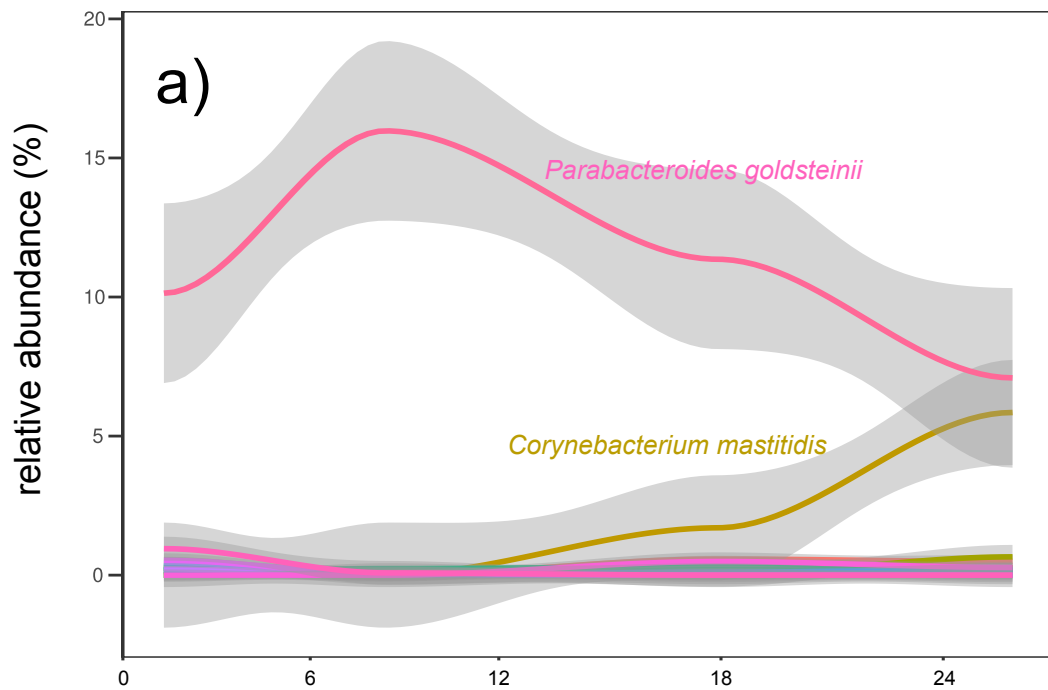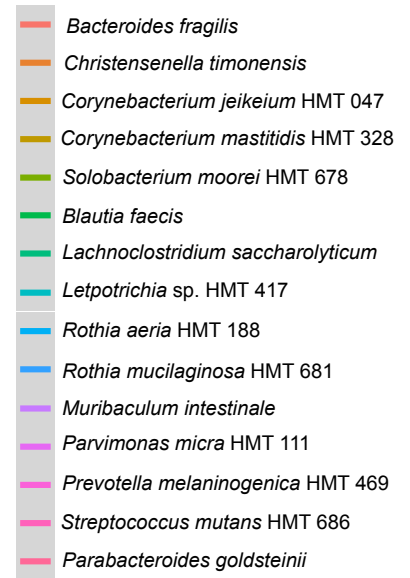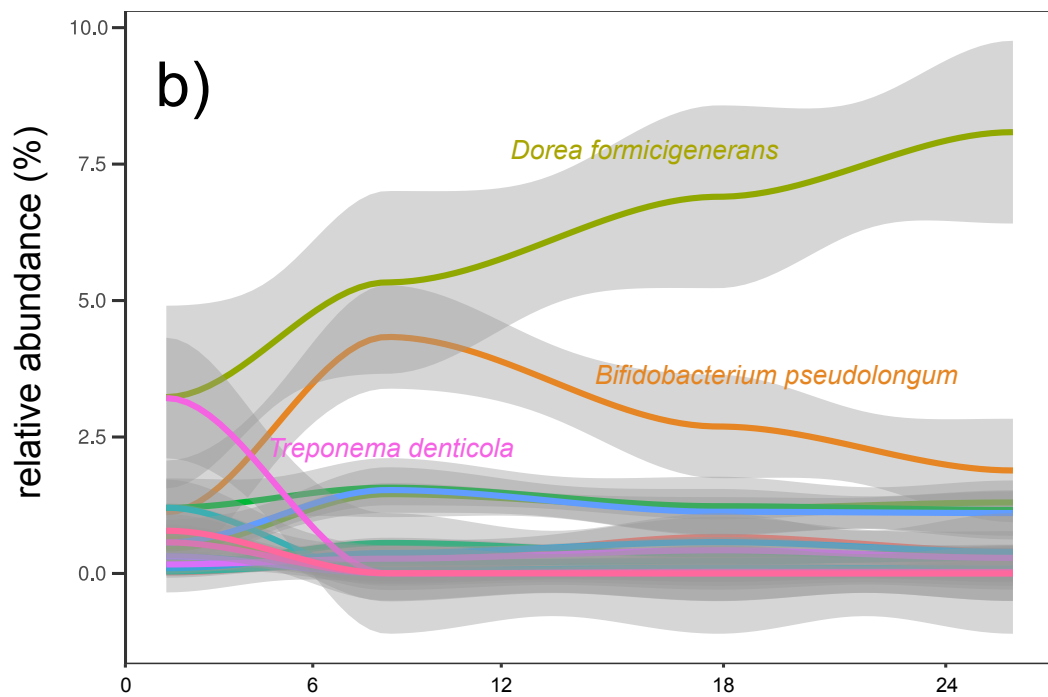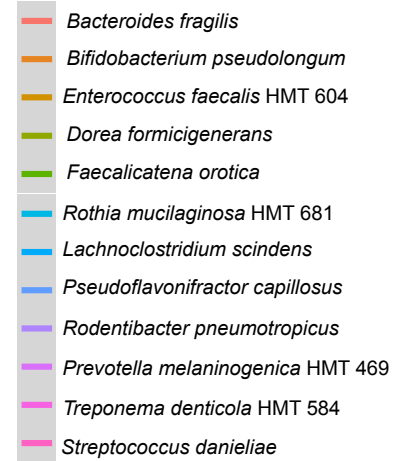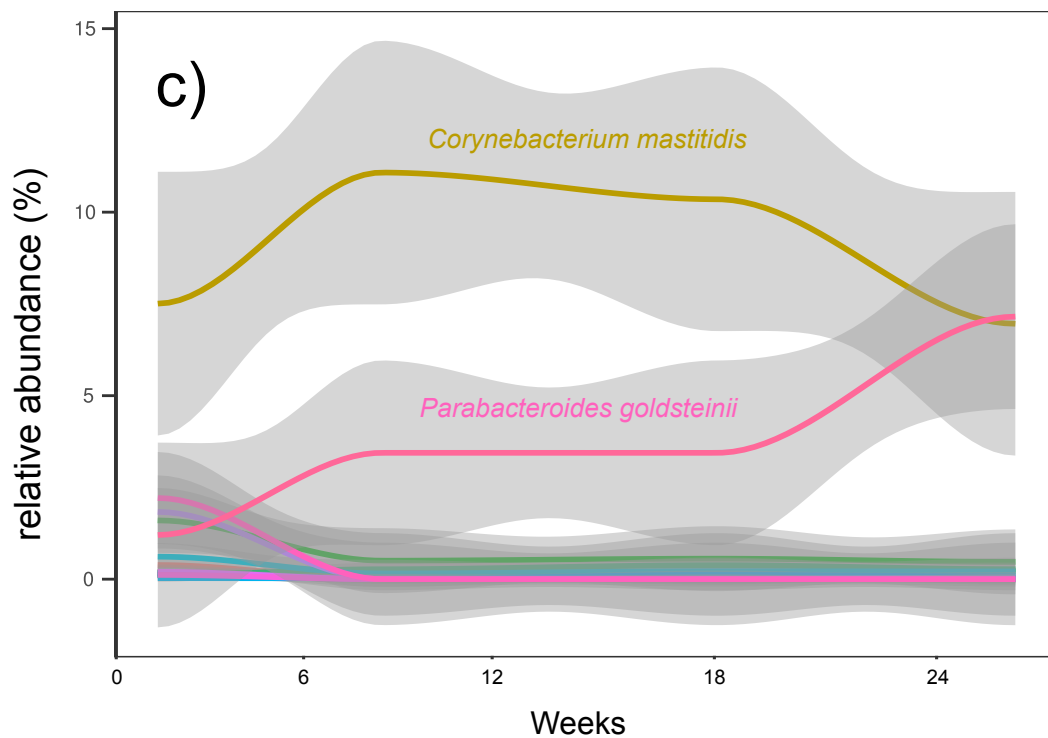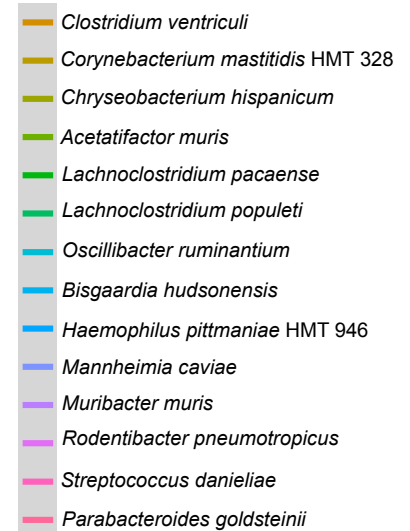

Supplement: FIG S4 [file mSystems.00323-19-sf004.pdf]

— Control  
— OSCC

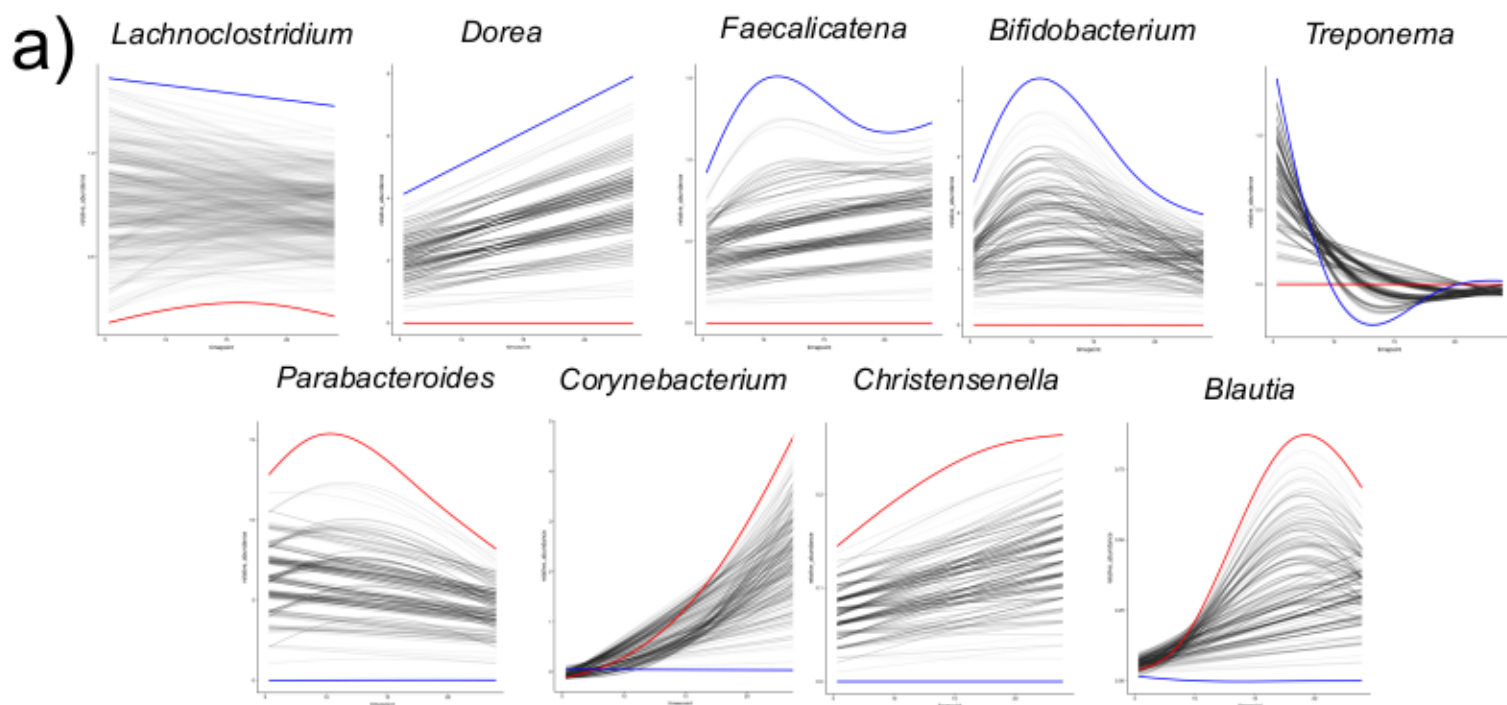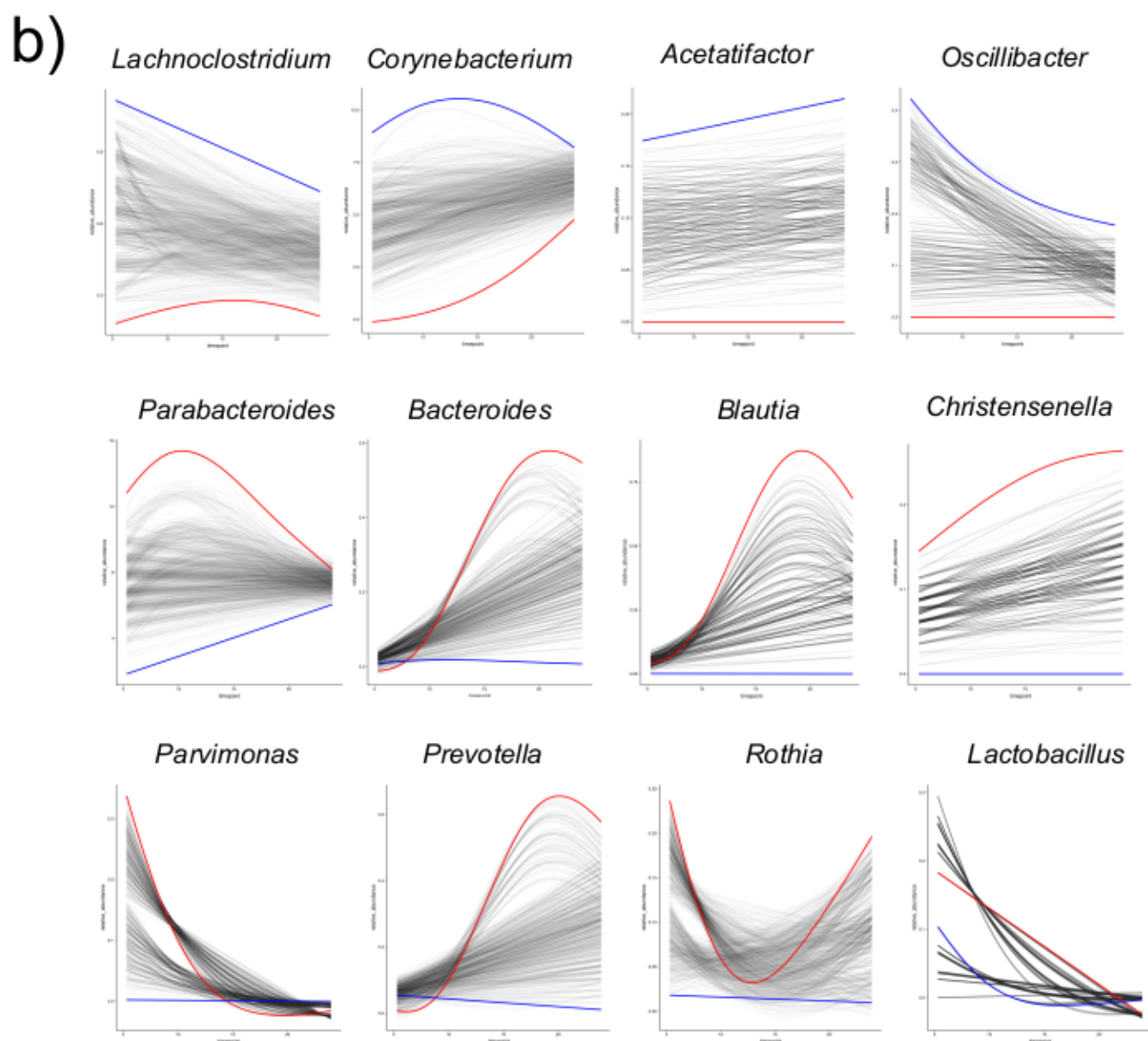

Supplement: FIG S5 [file mSystems.00323-19-sf005.pdf]

Control  
Group 3  
Group 4

a)

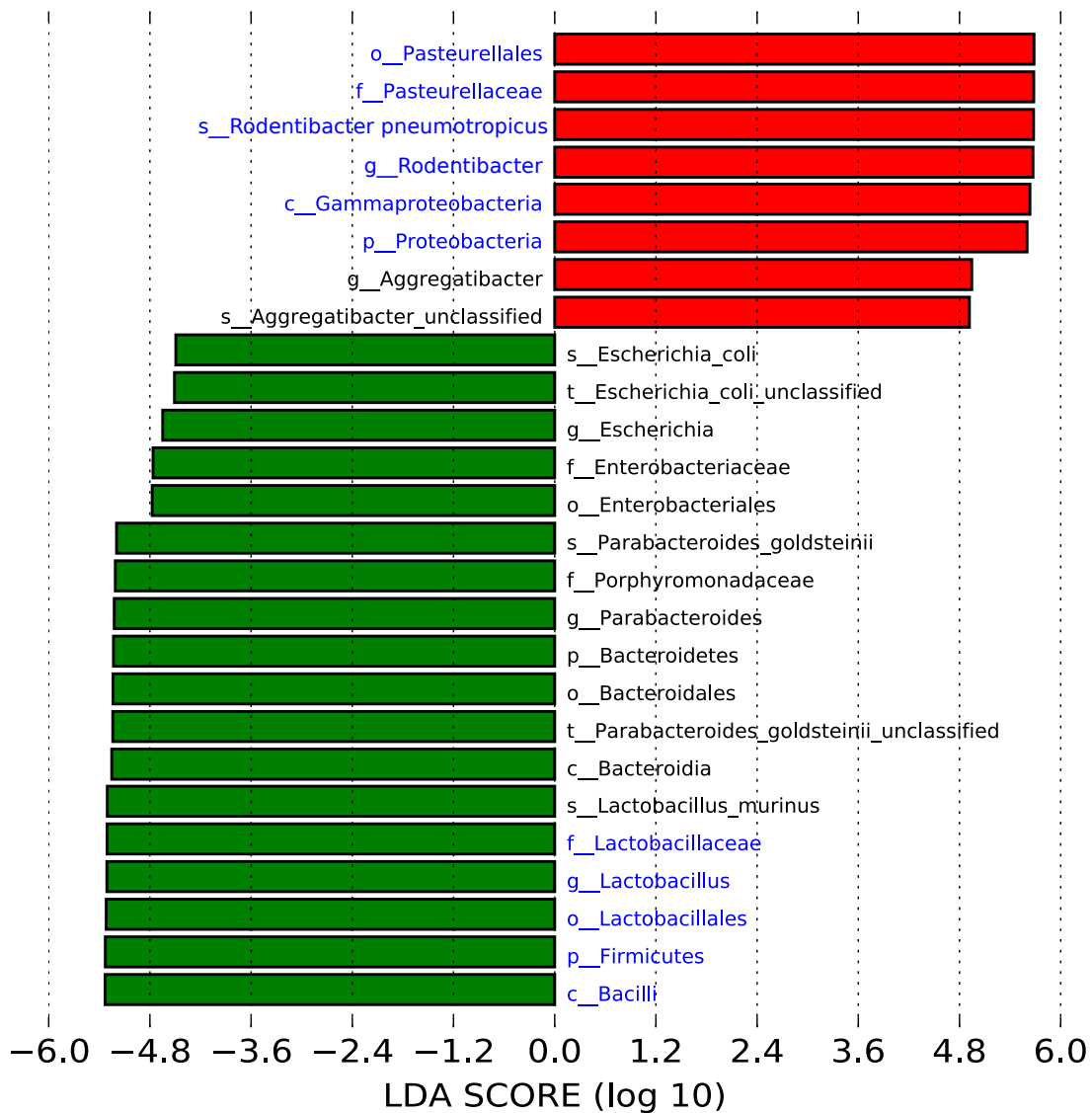

b)

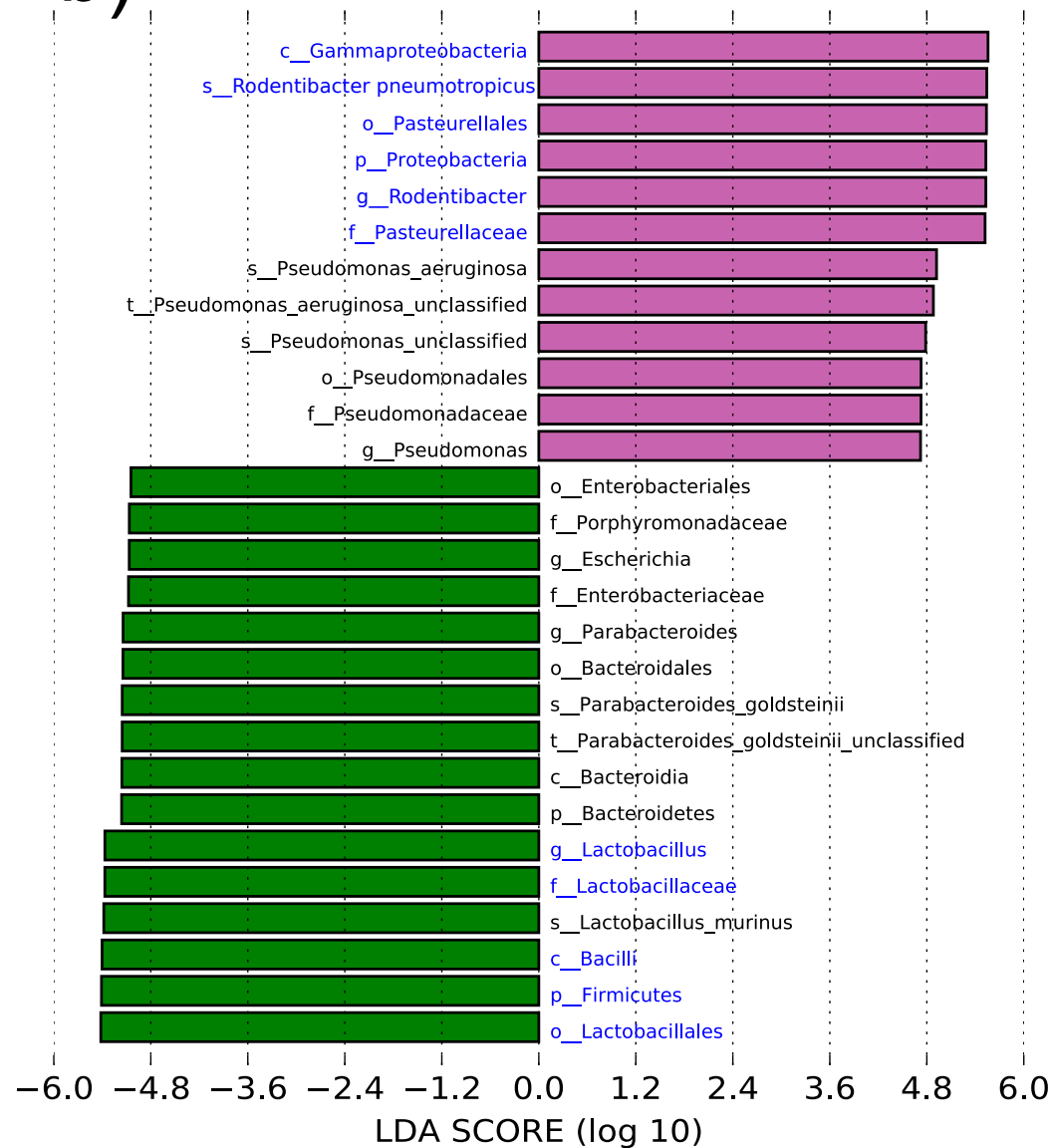

Supplement: FIG S7 [file mSystems.00323-19-sf007.pdf]
