# Supplementary material for: The Oral Mouse Microbiome Promotes Tumorigenesis in Oral Squamous Cell Carcinoma
Source: mSystems. 2019 Aug 6;4(4):e00323-19. doi: 10.1128/mSystems.00323-19 (PMC6687944; doi:10.1128/mSystems.00323-19)
Supplement: FIG S6 [file mSystems.00323-19-sf006.pdf]

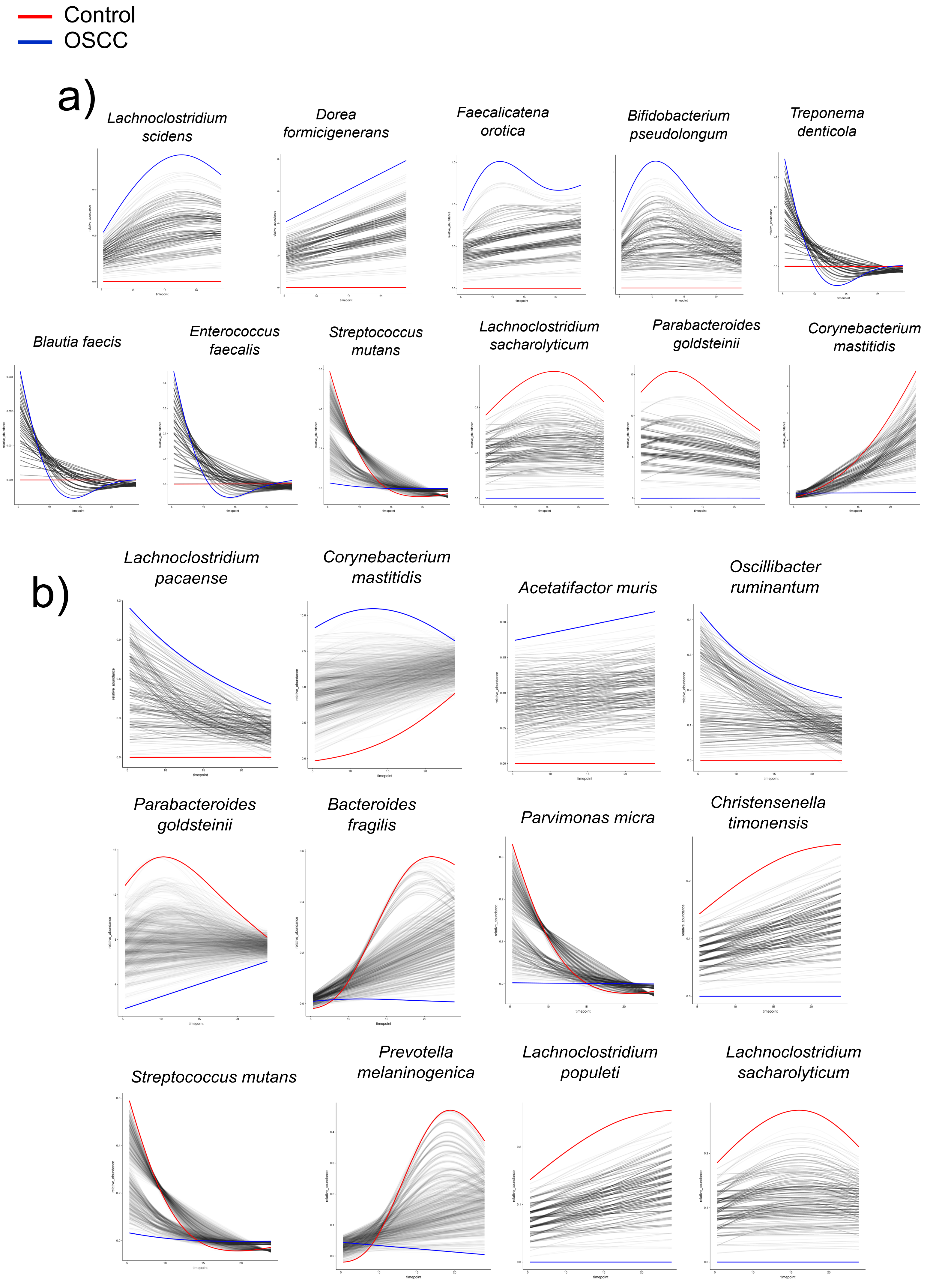
Control
OSCC
a)
Lachnoclostridium scidens
Dorea formicigenerans
Faecalicatena orotica
Bifidobacterium pseudolongum
Treponema denticola
Blautia faecis
Enterococcus faecalis
Streptococcus mutans
Lachnoclostridium sacharolyticum
Parabacteroides goldsteinii
Corynebacterium mastitidis
b)
Lachnoclostridium pacaense
Corynebacterium mastitidis
Acetatifactor muris
Oscillibacter ruminantum
Parabacteroides goldsteinii
Bacteroides fragilis
Parvimonas micra
Christensenella timonensis
Streptococcus mutans
Prevotella melaninogenica
Lachnoclostridium populeti
Lachnoclostridium sacharolyticum
