# Supplementary material for: The Oral Mouse Microbiome Promotes Tumorigenesis in Oral Squamous Cell Carcinoma
Source: mSystems. 2019 Aug 6;4(4):e00323-19. doi: 10.1128/mSystems.00323-19 (PMC6687944; doi:10.1128/mSystems.00323-19)
Supplement: TABLE S1 [file mSystems.00323-19-st001.pdf]

Supplementary Table 1. Statistical significance of the differences in trajectories for the 20 most abundant genera and species. Table shows the FDR ajusted-p values (q-values) of the different tests performed.

Control vs. Group 3

Genera	FDR Adjusted p-value
p: Actinobacteria.c: Actinobacteria.o: Actinomycetales.f: Micrococcaceae.g: Rothia	0.105
**p: Actinobacteria.c: Actinobacteria.o: Bifidobacteriales.f: Bifidobacteriaceae.g: Bifidobacterium**	**0.001**
**p: Actinobacteria.c: Actinobacteria.o: Corynebacteriales.f: Corynebacteriaceae.g: Corynebacterium**	**0.002**
p: Bacteroidetes.c: Bacteroidia.o: Bacteroidales.f: Bacteroidaceae.g: Bacteroides	0.75
p: Bacteroidetes.c: Bacteroidia.o: Bacteroidales.f: Prevotellaceae.g: Prevotella	0.298
**p: Bacteroidetes.c: Bacteroidia.o: Bacteroidales.f: Tannerellaceae.g: Parabacteroides**	**0.003**
p: Firmicutes.c: Bacilli.o: Lactobacillales.f: Enterococcaceae.g: Enterococcus	0.066
p: Firmicutes.c: Bacilli.o: Lactobacillales.f: Lactobacillaceae.g: Lactobacillus	0.992
p: Firmicutes.c: Bacilli.o: Lactobacillales.f: Streptococcaceae.g: Streptococcus	0.678
**p: Firmicutes.c: Clostridia.o: Clostridiales.f: Christensenellaceae.g: Christensenella**	**0.001**
**p: Firmicutes.c: Clostridia.o: Clostridiales.f: Lachnospiraceae.g: Blautia**	**0.001**
**p: Firmicutes.c: Clostridia.o: Clostridiales.f: Lachnospiraceae.g: Dorea**	**0.001**
**p: Firmicutes.c: Clostridia.o: Clostridiales.f: Lachnospiraceae.g: Faecalicatena**	**0.001**
**p: Firmicutes.c: Clostridia.o: Clostridiales.f: Lachnospiraceae.g: Lachnoclostridium**	**0.002**
**p: Firmicutes.c: Clostridia.o: Clostridiales.f: multifamily.g: Lachnoclostridium**	**0.001**
**p: Firmicutes.c: Clostridia.o: Clostridiales.f: multifamily.g: Pseudoflavonifractor**	**0.004**
p: Fusobacteria.c: Fusobacteriia.o: Fusobacteriales.f: Fusobacteriaceae.g: Fusobacterium	0.282
**p: Spirochaetes.c: Spirochaetia.o: Spirochaetales.f: Spirochaetaceae.g: Treponema**	**0.002**
**Species**	
p: Actinobacteria.c: Actinobacteria.o: Actinomycetales.f: Micrococcaceae.g: Rothia mucilaginosa HMT 681	0.098
**p: Actinobacteria.c: Actinobacteria.o: Bifidobacteriales.f: Bifidobacteriaceae.g: Bifidobacterium pseudolongum**	**0.001**
**p: Actinobacteria.c: Actinobacteria.o: Corynebacteriales.f: Corynebacteriaceae.g: Corynebacterium mastitidis HMT 328**	**0.002**
p: Bacteroidetes.c: Bacteroidia.o: Bacteroidales.f: Bacteroidaceae.g: Bacteroides fragilis	0.708
p: Bacteroidetes.c: Bacteroidia.o: Bacteroidales.f: Prevotellaceae.g: Prevotella melaninogenica HMT 469	0.468
**p: Bacteroidetes.c: Bacteroidia.o: Bacteroidales.f: Tannerellaceae.g: Parabacteroides goldsteinii**	**0.002**
**p: Firmicutes.c: Bacilli.o: Lactobacillales.f: Enterococcaceae.g: Enterococcus faecalis HMT 604**	**0.043**
**p: Firmicutes.c: Bacilli.o: Lactobacillales.f: Streptococcaceae.g: Streptococcus mutans HMT 686**	**0.002**
**p: Firmicutes.c: Clostridia.o: Clostridiales.f: Lachnospiraceae.g: Blautia faecis**	**0.023**
**p: Firmicutes.c: Clostridia.o: Clostridiales.f: Lachnospiraceae.g: Dorea formicigenerans**	**0.001**
**p: Firmicutes.c: Clostridia.o: Clostridiales.f: Lachnospiraceae.g: Faecalicatena orotica**	**0.001**
**p: Firmicutes.c: Clostridia.o: Clostridiales.f: Lachnospiraceae.g: Lachnoclostridium saccharolyticum**	**0.001**
**p: Firmicutes.c: Clostridia.o: Clostridiales.f: multifamily.g: Lacnoclostridium scindens**	**0.001**
**p: Firmicutes.c: Clostridia.o: Clostridiales.f: multifamily.g: Pseudoflavonifractor capillosus**	**0.003**
**p: Spirochaetes.c: Spirochaetia.o: Spirochaetales.f: Spirochaetaceae.g: Treponema denticola HMT 584**	**0.002**

Supplementary Table 1 (cont). Statistical significance of the differences in trajectories for the 20 most abundant genera and species. Table shows the FDR ajusted-p values (q-values) of the different tests performed.

Control vs. Group 4

Genera	FDR Adjusted p-value
**p: Actinobacteria.c: Actinobacteria.o: Actinomycetales.f: Micrococcaceae.g: Rothia**	**0.05**
**p: Actinobacteria.c: Actinobacteria.o: Corynebacteriales.f: Corynebacteriaceae.g: Corynebacterium**	**0.001**
**p: Bacteroidetes.c: Bacteroidia.o: Bacteroidales.f: Bacteroidaceae.g: Bacteroides**	**0.003**
**p: Bacteroidetes.c: Bacteroidia.o: Bacteroidales.f: Prevotellaceae.g: Prevotella**	**0.009**
**p: Bacteroidetes.c: Bacteroidia.o: Bacteroidales.f: Tannerellaceae.g: Parabacteroides**	**0.001**
**p: Firmicutes.c: Bacilli.o: Lactobacillales.f: Lactobacillaceae.g: Lactobacillus**	**0.013**
p: Firmicutes.c: Bacilli.o: Lactobacillales.f: Streptococcaceae.g: Streptococcus	0.482
**p: Firmicutes.c: Clostridia.o: Clostridiales.f: Christensenellaceae.g: Christensenella**	**0.002**
**p: Firmicutes.c: Clostridia.o: Clostridiales.f: Lachnospiraceae.g: Acetatifactor**	**0.002**
**p: Firmicutes.c: Clostridia.o: Clostridiales.f: Lachnospiraceae.g: Blautia**	**0.002**
**p: Firmicutes.c: Clostridia.o: Clostridiales.f: Lachnospiraceae.g: Lachnoclostridium**	**0.003**
**p: Firmicutes.c: Clostridia.o: Clostridiales.f: Oscillospiraceae.g: Oscillibacter**	**0.002**
**p: Firmicutes.c: Clostridia.o: Clostridiales.f: Peptoniphilaceae.g: Parvimonas**	**0.003**
p: Fusobacteria.c: Fusobacteriia.o: Fusobacteriales.f: Fusobacteriaceae.g: Fusobacterium	0.418
p: Fusobacteria.c: Fusobacteriia.o: Fusobacteriales.f: Leptotrichiaceae.g: Leptotrichia	0.279
p: Proteobacteria.c: Gammaproteobacteria.o: Pasteurellales.f: Pasteurellaceae.g: Muribacter	0.071
**Species**	
**p: Actinobacteria.c: Actinobacteria.o: Corynebacteriales.f: Corynebacteriaceae.g: Corynebacterium mastitidis HMT 328**	**0.002**
**p: Bacteroidetes.c: Bacteroidia.o: Bacteroidales.f: Bacteroidaceae.g: Bacteroides fragilis**	**0.001**
**p: Bacteroidetes.c: Bacteroidia.o: Bacteroidales.f: Prevotellaceae.g: Prevotella melaninogenica HMT 469**	**0.005**
**p: Firmicutes.c: Bacilli.o: Lactobacillales.f: Streptococcaceae.g: Streptococcus danieliae**	**0.082**
**p: Firmicutes.c: Bacilli.o: Lactobacillales.f: Streptococcaceae.g: Streptococcus mutans HMT 686**	**0.001**
**p: Firmicutes.c: Clostridia.o: Clostridiales.f: Christensenellaceae.g: Christensenella timonensis**	**0.001**
**p: Firmicutes.c: Clostridia.o: Clostridiales.f: Lachnospiraceae.g: Acetatifactor muris**	**0.002**
p: Firmicutes.c: Clostridia.o: Clostridiales.f: Lachnospiraceae.g: Blautia faecis	1
**p: Firmicutes.c: Clostridia.o: Clostridiales.f: Lachnospiraceae.g: Lachnoclostridium pacaense**	**0.002**
**p: Firmicutes.c: Clostridia.o: Clostridiales.f: Lachnospiraceae.g: Lachnoclostridium populeti**	**0.001**
**p: Firmicutes.c: Clostridia.o: Clostridiales.f: Lachnospiraceae.g: Lachnoclostridium saccharolyticum**	**0.003**
**p: Firmicutes.c: Clostridia.o: Clostridiales.f: Oscillospiraceae.g: Oscillibacter ruminantium**	**0.001**
**p: Firmicutes.c: Clostridia.o: Clostridiales.f: Peptoniphilaceae.g: Parvimonas micra HMT 111**	**0.002**
p: Proteobacteria.c: Gammaproteobacteria.o: Pasteurellales.f: Pasteurellaceae.g: Muribacter muris	0.07
