# Supplementary material for: The Oral Mouse Microbiome Promotes Tumorigenesis in Oral Squamous Cell Carcinoma
Source: mSystems. 2019 Aug 6;4(4):e00323-19. doi: 10.1128/mSystems.00323-19 (PMC6687944; doi:10.1128/mSystems.00323-19)

66 common elements in "Group3" and "Group4":

(5Z)-dodec-5-enoate biosynthesis
5-aminoimidazole ribonucleotide biosynthesis I
5-aminoimidazole ribonucleotide biosynthesis II
ADP-L-glycero-β-D-manno-heptose biosynthesis
aerobic respiration II (cytochrome c) (yeast)
C4 photosynthetic carbon assimilation cycle, PEPCK type
Calvin-Benson-Bassham cycle
CDP-diacylglycerol biosynthesis I
CDP-diacylglycerol biosynthesis II
CMP-3-deoxy-D-manno-octulosonate biosynthesis I
fatty acid elongation -- saturated
gluconeogenesis I
glycolysis I (from glucose 6-phosphate)
glycolysis II (from fructose 6-phosphate)
gondoate biosynthesis (anaerobic)
inosine-5'-phosphate biosynthesis I
inosine-5'-phosphate biosynthesis II
inosine-5'-phosphate biosynthesis III
L-histidine biosynthesis
L-homoserine and L-methionine biosynthesis
L-isoleucine biosynthesis I
L-isoleucine biosynthesis III
L-lysine biosynthesis I
L-lysine biosynthesis III
L-lysine biosynthesis VI
L-methionine biosynthesis I
L-valine biosynthesis
mixed acid fermentation
myo-, chiro- and scillo-inositol degradation
N10-formyl-tetrahydrofolate biosynthesis
NAD/NADH phosphorylation and dephosphorylation
octanoyl-[acyl-carrier protein] biosynthesis (mitochondria, yeast)
oleate biosynthesis IV (anaerobic)
palmitoleate biosynthesis I (from (5Z)-dodec-5-enoate)
pentose phosphate pathway
pentose phosphate pathway (non-oxidative branch)
phosphatidylglycerol biosynthesis I (plastidic)
phosphatidylglycerol biosynthesis II (non-plastidic)
pyrimidine deoxyribonucleotides de novo biosynthesis II
pyruvate fermentation to acetate and lactate II
pyruvate fermentation to isobutanol (engineered)
S-adenosyl-L-methionine cycle I
seleno-amino acid biosynthesis
stearate biosynthesis II (bacteria and plants)
superpathway of 5-aminoimidazole ribonucleotide biosynthesis
superpathway of acetyl-CoA biosynthesis
superpathway of adenosine nucleotides de novo biosynthesis I
superpathway of adenosine nucleotides de novo biosynthesis II
superpathway of branched amino acid biosynthesis
superpathway of fatty acid biosynthesis initiation (E. coli)
superpathway of glucose and xylose degradation
superpathway of L-alanine biosynthesis
superpathway of L-aspartate and L-asparagine biosynthesis
superpathway of L-isoleucine biosynthesis I
superpathway of L-lysine, L-threonine and L-methionine biosynthesis II
superpathway of L-methionine biosynthesis (transsulfuration)
superpathway of L-serine and glycine biosynthesis I
superpathway of L-threonine biosynthesis
superpathway of N-acetylglucosamine, N-acetylmannosamine and N-acetylneuraminate degradation
superpathway of phospholipid biosynthesis I (bacteria)
superpathway of purine nucleotides de novo biosynthesis II
superpathway of S-adenosyl-L-methionine biosynthesis
TCA cycle VI (obligate autotrophs)
tRNA charging
tRNA processing
urate biosynthesis/inosine 5'-phosphate degradation

9 elements included exclusively in "Group 3":

6-hydroxymethyl-dihydropterin diphosphate biosynthesis I
6-hydroxymethyl-dihydropterin diphosphate biosynthesis III
adenosine deoxyribonucleotides de novo biosynthesis II
flavin biosynthesis III (fungi)
guanosine deoxyribonucleotides de novo biosynthesis II
L-tryptophan biosynthesis
NAD salvage pathway II
peptidoglycan maturation (meso-diaminopimelate containing)
queuosine biosynthesis

5 elements included exclusively in "Group4":

folate transformations II
pyrimidine deoxyribonucleotides de novo biosynthesis I
superpathway of purine nucleotides de novo biosynthesis I
superpathway of pyrimidine deoxyribonucleotides de novo biosynthes[illegible]
tetrapyrrole biosynthesis I (from glutamate)

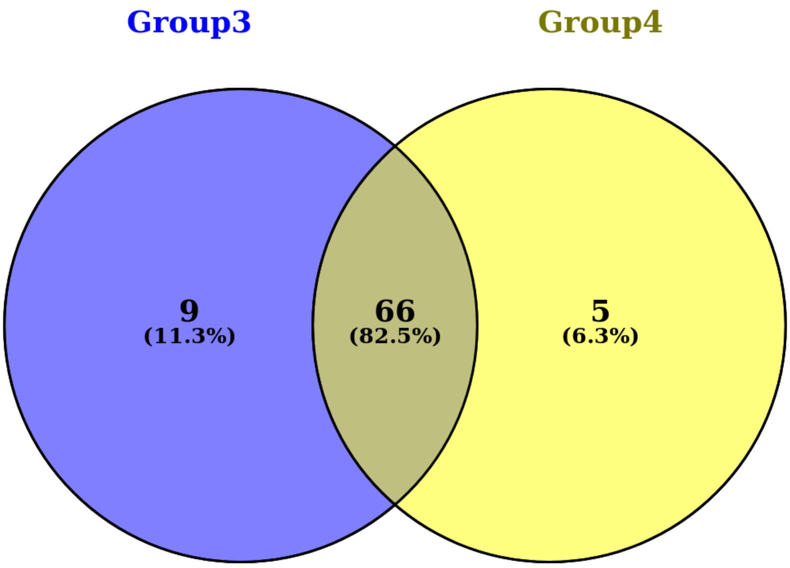

Supplement: FIG S8 [file mSystems.00323-19-sf008.pdf]
